# Hypoplastic left heart syndrome: anatomical spectrum, clinical management, and brain defects

**DOI:** 10.3389/fcvm.2026.1877717

**Published:** 2026-10-29

**Authors:** Yolandee Bell-Cheddar, William Devine, Carlos Eduardo Diaz-Castrillon, Richard Tang, Mario Castro-Medina, Shannon A. Solt, Adam Christopher, Raymond Morales, XinXiu Xu, Cecilia W. Lo, Jiuann-Huey I. Lin

**Affiliations:** 1 Department of Critical Care Medicine, University of Pittsburgh School of Medicine, Pittsburgh, PA, United States; 2 Department of Pediatrics, UPMC Children’s Hospital of Pittsburgh, Pittsburgh, PA, United States; 3 Department of Cardiothoracic Surgery, UPMC Children's Hospital of Pittsburgh, Pittsburgh, PA, United States

**Keywords:** cardiomyocyte, complex genetics, hemodynamics, HLHS, mitochondria, single ventricle, surgical interventions

## Abstract

Hypoplastic left heart syndrome (HLHS) is a complex congenital heart disease (CHD) involving hypoplasia of the left ventricle (LV) and the aorta (Ao), along with aortic and mitral valve stenosis/atresia with or without coarctation. It is a physiologically single-ventricle lesion, previously uniformly fatal, but now survivable with a 3-stage surgical palliation ending with the Fontan circulation, a circulation for only one working ventricle. In the classical definition of HLHS, the ventricular septum is intact; however, HLHS variants have been described with similar single-ventricle pathophysiology in which the hypoplastic LV is not hemodynamically coupled to the aortic outflow. Evidence for a genetic etiology for HLHS is indicated by its high recurrence risk, the association with various chromosomal abnormalities, and the demonstration of mutations in mice causing HLHS. Despite surgical advances allowing survival of patients with HLHS, there is still high morbidity and mortality after their first-stage palliation. The causes of such high morbidity and mortality are not well understood, but most deaths are likely related to cardiovascular/hemodynamic causes. Studies in the animal models of HLHS show that this cardiac lesion arises early in embryogenesis, decreases left-sided inflow, and is associated with metabolic dysfunction, cell proliferation, and differentiation defects. Findings indicate HLHS is also associated with intrinsic cell deficits, deficits that may also contribute to the high risk of heart failure and adverse neurodevelopment. In this article, we update a previous review by describing the basic pathophysiology; pertinent pre- and postoperative management of HLHS and its anatomical variants; and novel insights from the HLHS mouse model on the causes of HLHS-associated heart failure and poor neurodevelopment, as well as potential therapeutic strategies.

## 1 Introduction

Hypoplastic left heart syndrome (HLHS) is a complex cyanotic congenital heart defect involving hypoplasia of the left ventricle (LV), mitral valve (MV), and aorta (AO), with variants displaying different degrees of left-sided hypoplasia and outflow tract obstructions, which are critical for accurate diagnosis and management (1, 2) (Figure 1). Although HLHS constitutes 2%-3% of all congenital heart defects, it accounts for 25% of CHD infant deaths (1), and it remains the most challenging to achieve a good outcome in a sustainable fashion (3, 4).

**Figure 1:**
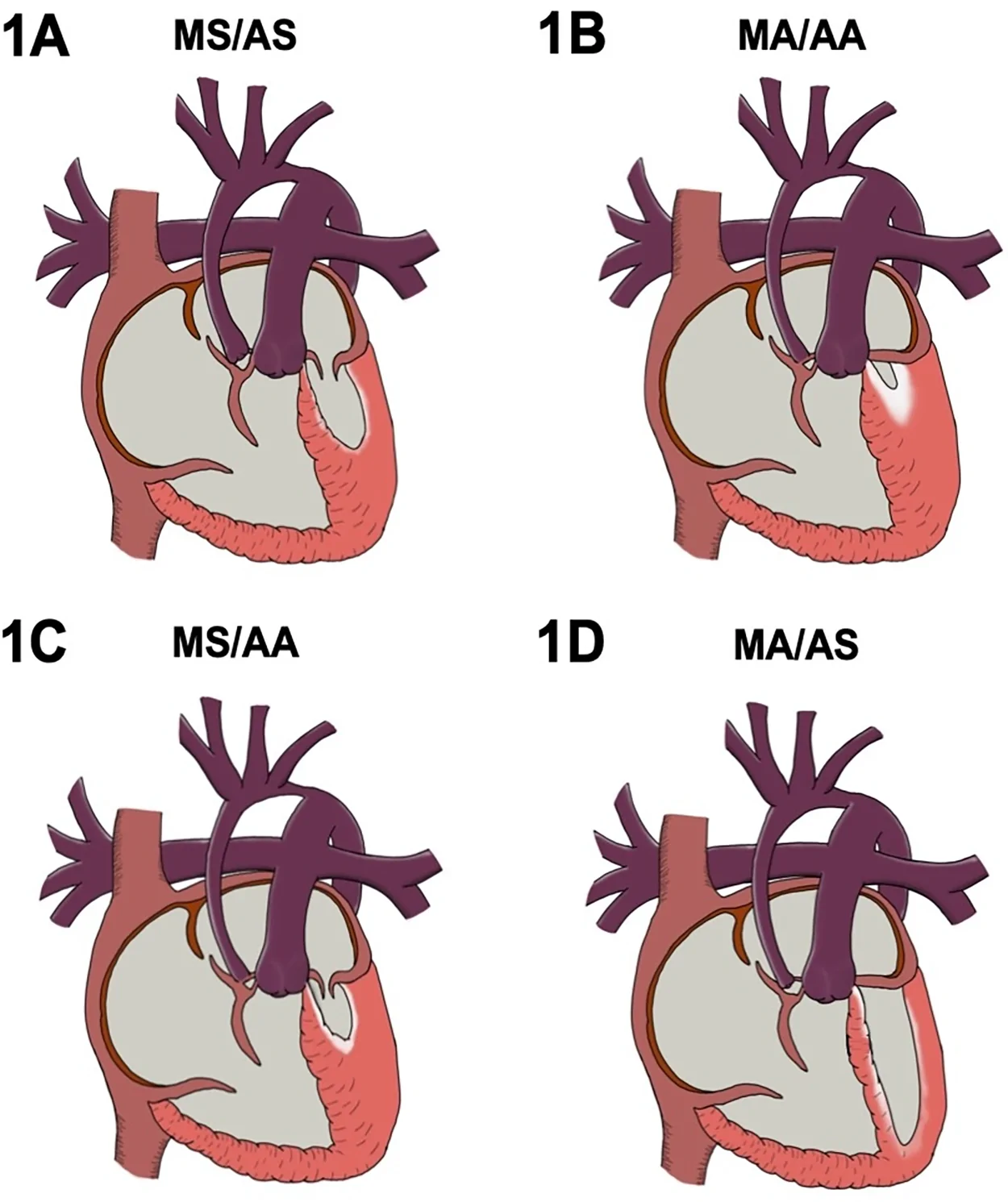
Illustration of 4 types of HLHS (graphic illustration by Dr. Raymond Morales) [with permission from Bell-Cheddar et al. (2)]. AA, aortic atresia; AS, aortic stenosis; MA, mitral atresia; MS, mitral stenosis. Modified with permission from Bell-Cheddar et al. (2).

Significant surgical advancements in HLHS have transformed a once uniformly fatal defect before the 1980s into one that is now survivable with a three-stage cardiac surgical palliation or with cardiac transplantation. The reported 54%–59% transplant-free survival at 12 years after the Norwood operation is still suboptimal (4). Most HLHS survivors suffer ongoing morbidity, with a substantial portion of these patients developing heart failure over time, as well as neurodevelopmental delay and neurocognitive impairment that can significantly impact health-related quality of life. The causes for such high morbidity and mortality are not well understood, but most deaths are directly or indirectly related to cardiovascular causes (5). While the causes of poor cardiac outcomes are multifactorial (4), our study in a mouse model of HLHS identified important contributing factors in physiological single-ventricle patients related to mitochondrial dysfunction (6).

## 2 Section I: the morphologic spectrum of hypoplastic left heart syndrome

Dr. Maurice Lev first described the hypoplasia of the aortic tract as the hypoplastic left heart in 1952 (7). Noonan and Nadas initially used the term “hypoplastic left heart syndrome” in 1958 (8). HLHS shows a spectrum of left ventricular inlet, chamber, and outlet obstructions, including aortic atresia or stenosis, and mitral stenosis, or atresia, or agenesis (Figure 1). Glatz et al. reported a high-risk variant in patients with mitral stenosis/aortic atresia (HLHS MS/AA) compared with mitral atresia/aortic atresia and mitral stenosis/aortic stenosis (*P* = 0.015). However, aortic atresia alone was not associated with increased mortality (*P* = 0.2) (9). HLHS MS/AA variant potentially is related to ventriculo-coronary connections or endocardial fibroelastosis (EFE). Nevertheless, Wilson et al. reported the mortality or transplant prior to stage II palliation in 141 HLHS infants with MS/AA was not associated with ventriculo-coronary connections (*P* = 0.83) or EFE (*P* = 0.19) but was associated with the size of the ascending aorta (median, 2.2 vs. 2.6 mm, *P* = 0.01), birth weight (*P* = 0.003), gestational age (*P* < 0.0001) and longer cardiopulmonary bypass time (*P* = 0.001), longer cardiac intensive care unit length of stay (*P* = 0.001) and extracorporeal membrane oxygenation usage following Norwood procedure (*P* < 0.0001) (10).

### 2.1 Gross morphology of HLHS

A normal left ventricle (Figures 2A, 3A, 3E) has a mitral valve and connects to the aorta. The mitral valve is in fibrous continuity with the aortic valve (Figures 2A, 3A), and the subarterial septal surface is smooth (Figure 2A). Furthermore, the apical portion of the left ventricular septal surface shows smaller trabeculations than a morphologic right ventricle. Hearts with HLHS show variability in the degree of left ventricular hypoplasia (Figures 2B–E), the types of aortic valve malformations (Figures 3B–D) and the types of mitral valve obstruction (Figures 3F–H, 5C), the degree of ascending aorta and aortic arch hypoplasia (Figures 4A,B), and the presence or absence of a coarctation of the aorta (Figure 4B) (2).

**Figure 2:**
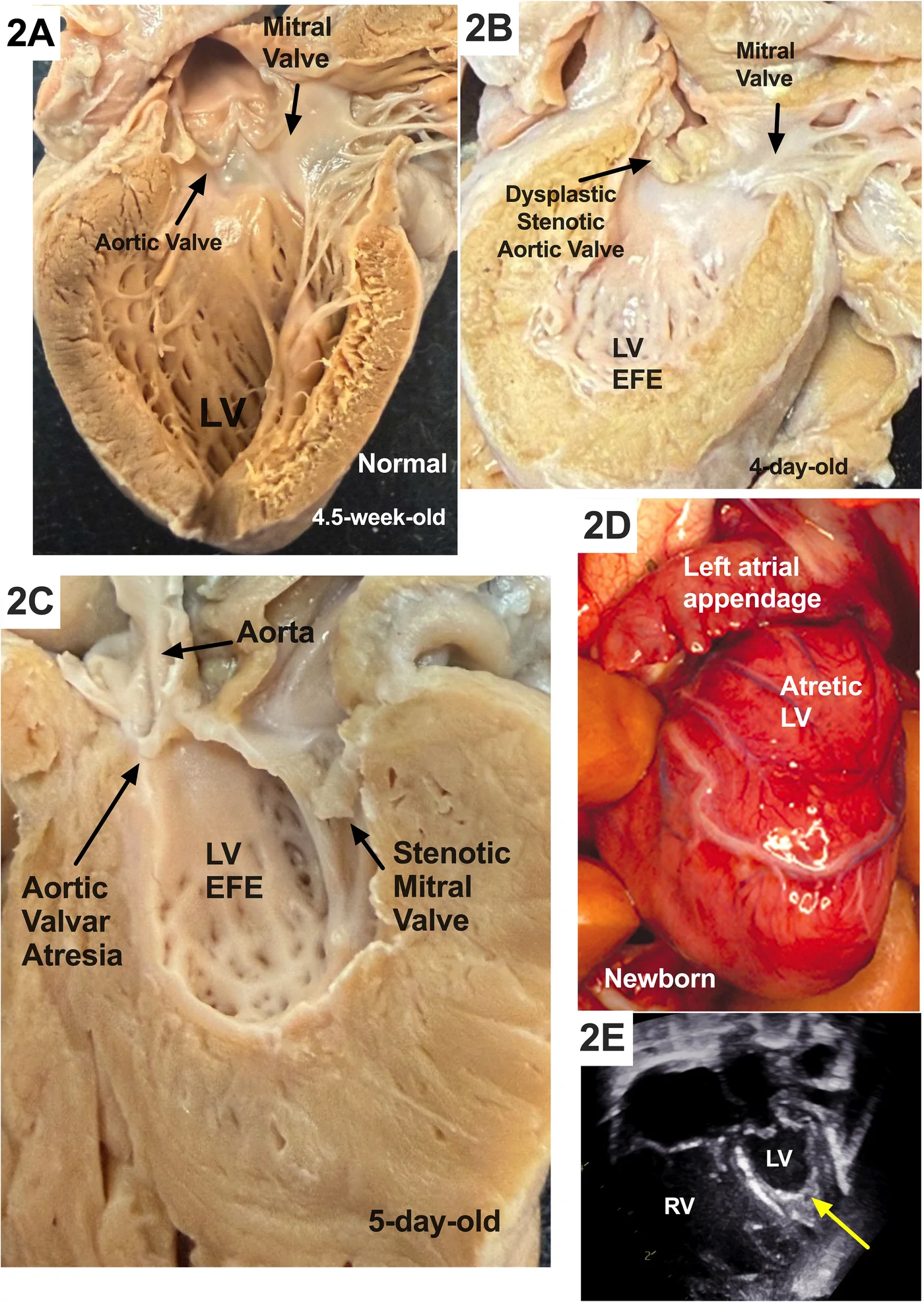
Variations in the degree of left ventricular hypoplasia in hearts with HLHS. **(A)** Septal view of a normal left ventricle, **(B)** hypoplastic left ventricle with a stenotic and dysplastic aortic valve and endocardial fibroelastosis, **(C)** shows the muscle-bound, non-apex-forming hypoplastic left ventricle with endocardial fibroelastosis, mitral valve stenosis and aortic valve atresia, **(D)** *in situ* image of a newborn infant with a hypoplastic left ventricle that has no inlet or outlet. The muscle-bound left ventricular segment of the heart bulges on the epicardial surface, and the anterior and posterior descending coronary arteries outline the position of the left ventricle. Subsequently, a minute atretic left ventricular chamber was identified. **(E)** TTE apical 4-chamber view showing hypoplastic left ventricle with endocardial fibroelastosis (arrows). LV: left ventricle.

**Figure 3:**
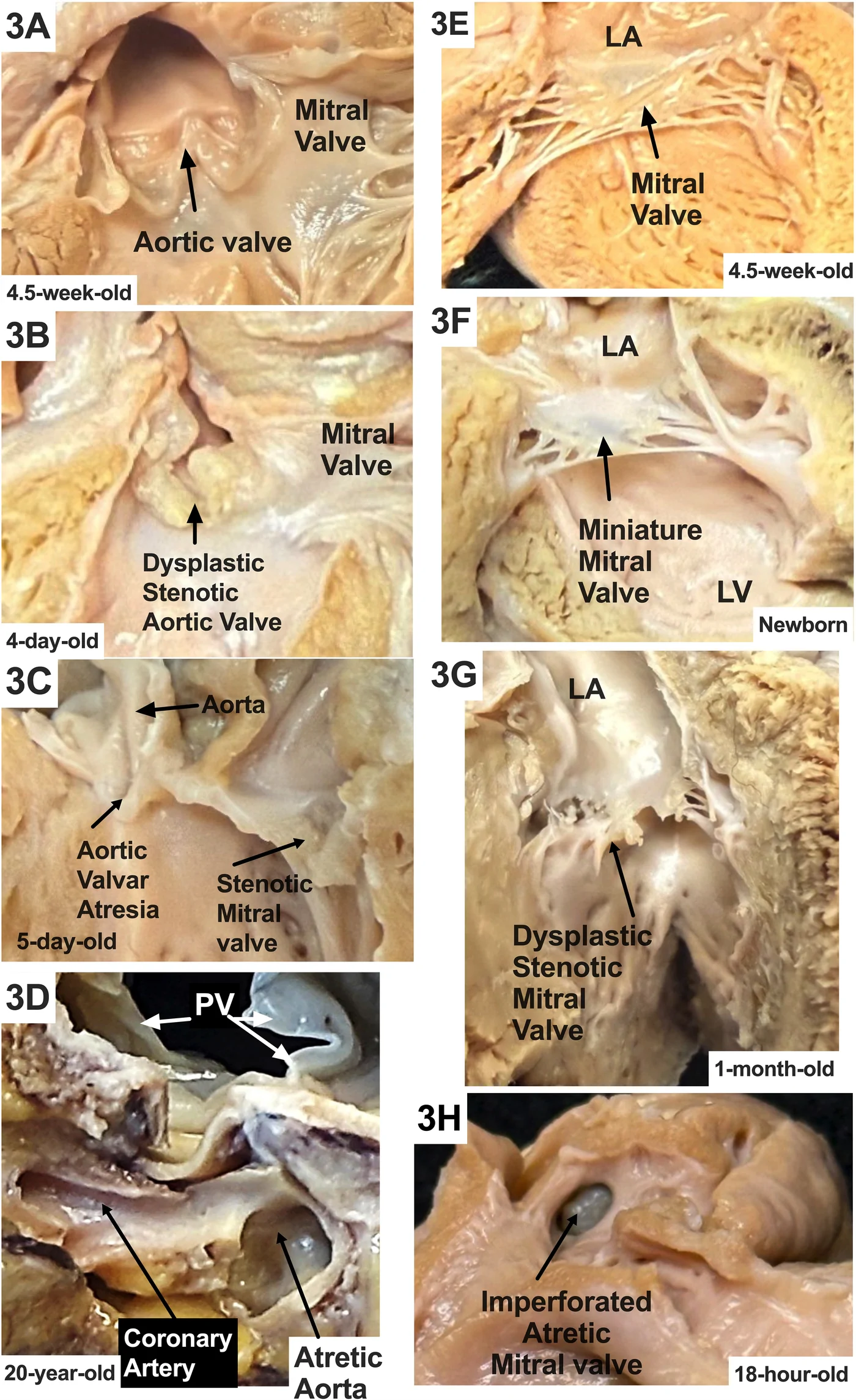
**Variations in the condition of the aortic and mitral valves in hearts with HLHS.** (**A**) normal aortic valve; (**B**) dysplastic, stenotic aortic valve; (**C**) aortic valvular membranous atresia, mitral valve stenosis, and endocardial fibroelastosis; and (**D**) base view of a cardiac explant from a 20-year-old, status post HLHS surgical palliation, showing a hypoplastic, blind-ending aortic root without leaflet tissue. (**E**) Normal mitral valve; (**F**) normally configured but miniaturized mitral valve with mildly thickened tension apparatus and diffuse endocardial fibroelastosis of the hypoplastic left ventricle; (**G**) stenotic and dysplastic mitral valve with endocardial fibroelastosis of the hypoplastic left ventricle; (**H**) floor of the left atrium and an imperforate mitral valve. LA: left atrium, LV: left ventricle.

**Figure 4:**
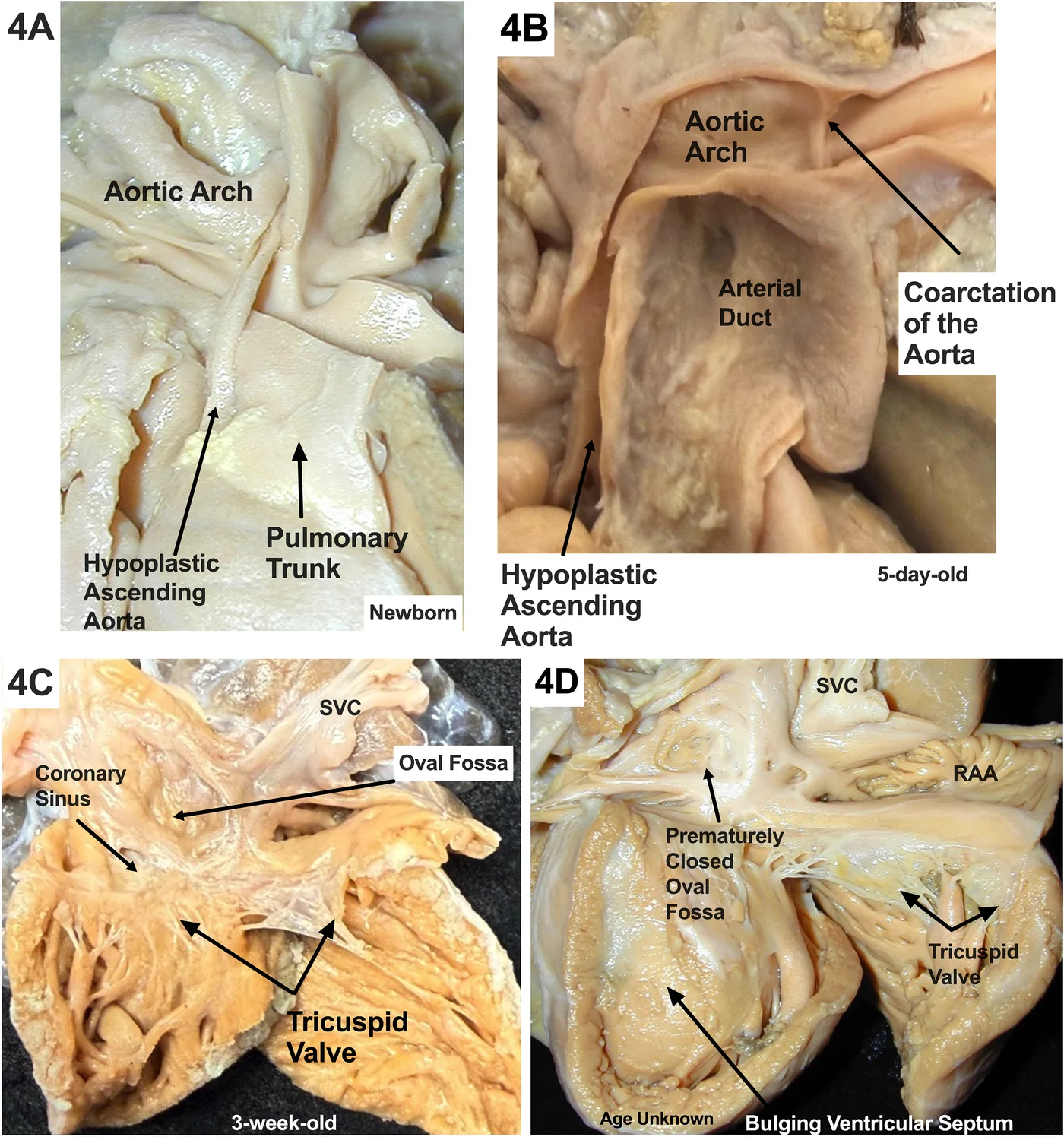
Great arteries of specimens showing HLHS and the right side of a specimen with HLHS- premature closure of the oval fossa. **(A)** very hypoplastic ascending aorta and large pulmonary trunk in a heart with HLHS, **(B)** hypoplastic ascending aorta and coarctation of the aorta at the level of a large arterial duct, **(C)** enlarged right heart in a patient with HLHS, **(D)** enlarged right heart showing HLHS-premature closure of the oval fossa with the muscle bound hypoplastic left ventricle causing the right side of the ventricular septum to bulge into the right ventricle. RAA, right atrial appendage; SVC, superior vena cava.

In hearts with classical HLHS, the hypoplastic left ventricle tends to be non-apex forming (2D, 5B), and the ventricular septum remains intact, but the left ventricular chamber can vary from diminutive and atretic with no inlet or outlet (Figure 2D) to milder forms of hypoplasia, with the left atrium showing corresponding degrees of hypoplasia. The hypoplastic left ventricle is often muscle-bound with a thick myocardial wall, causing bulging on the epicardial surface (Figure 2D). Endocardial fibroelastosis, which can be prominent, is also commonly observed in the hypoplastic left ventricle (Figures 2B,C,E) (2).

The normal mitral valve comprises anterior and posterior leaflets connected to papillary muscles (Figure 3E), with the anterior leaflet in fibrous continuity with the aortic valve (Figures 2A, 3A). In HLHS, the mitral valve can range from well-formed but miniature to dysplastic, stenotic, imperforate, or absent (Figures 3F–H, 5C), highlighting its variability.

The normal aortic valve consists of three semilunar cusps (Figures 2A, 3A), and the aorta consists of the aortic root with two coronary artery orifices, ascending aorta, aortic arch, isthmus, and descending aorta. Aortic valves in hearts with HLHS can vary from having three cusps to being bicuspid (Figure 5B) and are dysplastic, stenotic, or atretic (Figures 3B–D). The ascending aorta in HLHS may be mildly hypoplastic. Still, most of the time it is moderately to extremely hypoplastic, with an almost cord-like appearance (Figure 4A). Similarly, the aortic arch is often hypoplastic. Coarctation of the aorta, which is a shelf-like, circumferential periductal lesion obstructing with varying severity (Figure 4B), is also common in the setting of HLHS. Furthermore, HLHS is a ductal-dependent condition, and the arterial duct (PDA) will be widely patent and large (Figure 4B). As a result of the systemic dependence of the right heart as the functional pump, the right heart and pulmonary trunk will be enlarged (Figures 4A,C). An atrial communication with either a patent foramen ovale or an atrial septal defect is often present. Sometimes, the flap valve of the oval fossa can show aneurysmal dilatation bulging into the right atrium. At times, the muscle-bound hypoplastic left ventricle may cause the ventricular septum to bulge into the left ventricle, related to pressure differences or displacement during embryogenesis (Figure 4D).

**Figure 5:**
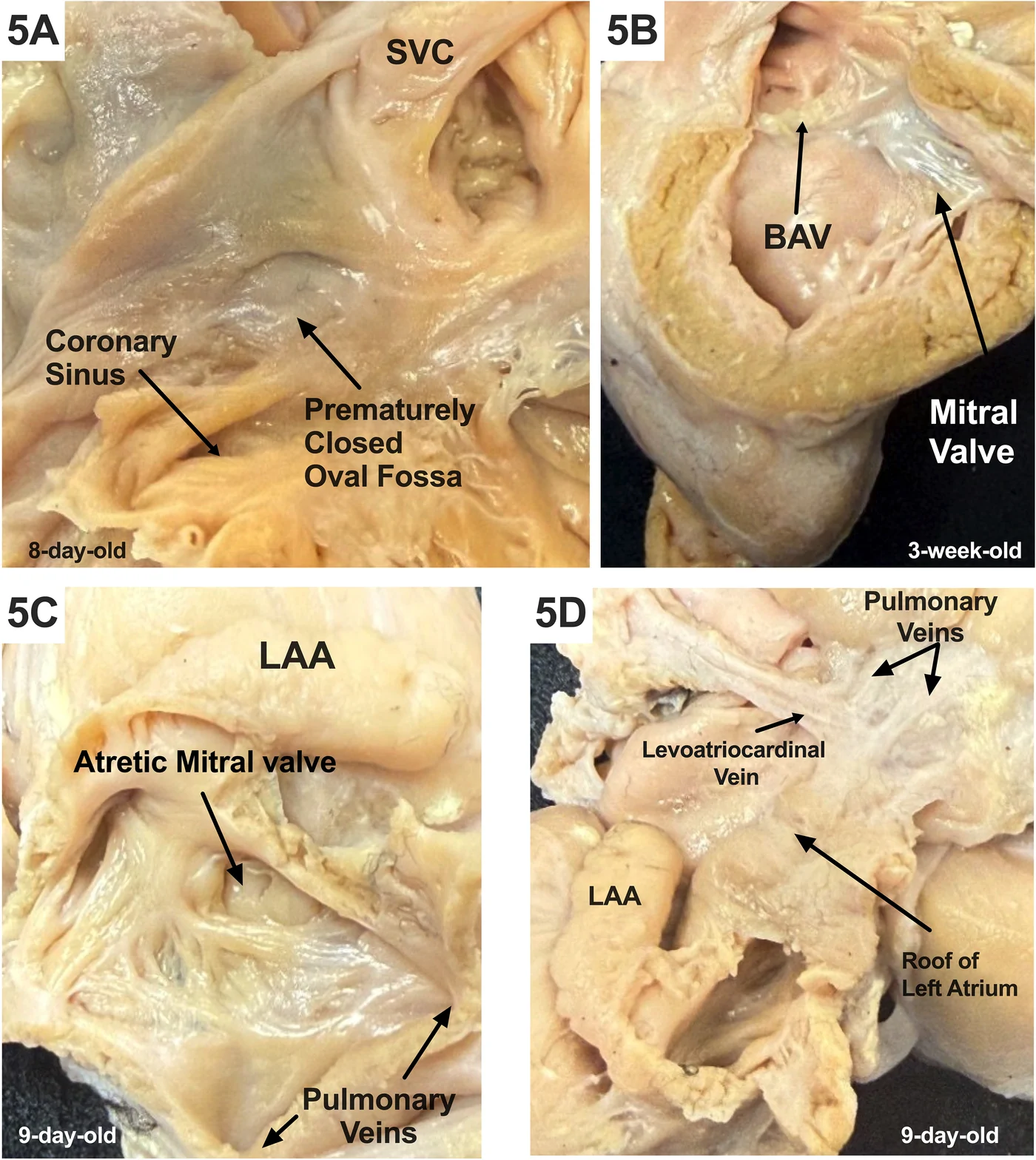
**Right and left heart anatomy of a heart with premature closure of the oval fossa, and a specimen with a levoatriocardinal vein with partial anomalous pulmonary venous return.** (**A**) septal surface of the right atrium illustrating the prematurely closed oval fossa, (**B**) non-apex-forming hypoplastic left ventricle with pronounced endocardial fibroelastosis and a bicuspid aortic valve, (**C**) floor of the left atrium of another heart showing agenesis (atresia) of the mitral valve, (**D**) a levoatriocardinal vein connecting the roof of the left atrium to the left innominate vein along with partial anomalous pulmonary venous return. BAV: bicuspid aortic valve, LAA: left atrial appendage, SVC: superior vena cava.

Hearts with premature closure of the oval fossa (Figures 4D, 5A) at an early gestational age yield decreased preload to the left heart, resulting in cardiac defects that constitute HLHS, with variable degrees of left ventricular hypoplasia, and endocardial fibroelastosis (EFE). The severity of the impact on the heart depends on the gestational timing of the closure or restriction of the oval fossa (11). Rarely, in patients born with premature closure of the oval fossa and inflow obstruction, such as mitral atresia, and in cases of typical HLHS with inflow obstruction, an escape channel for the pulmonary venous return may be present (12). This escape channel may be a levoatriocardinal vein (Figure 5D), which allows returned pulmonary venous blood to egress from the left atrium to reach the right atrium via the left innominate vein/superior caval vein (13). Infrequently, the escape of pulmonary venous blood from the left atrium may be accomplished by total or partial anomalous pulmonary venous return (Figure 5D) (14).

Simply having a hypoplastic left ventricle with aortic stenosis and an intact ventricular septum (Figure 6C) does not make that heart eligible to be classified as HLHS. Cardiac malformations such as a right dominant unbalanced atrioventricular septal defect (AVSD) (Figure 6D) or double outlet right ventricle (DORV) with mitral valve stenosis/atresia (Figures 6A,B), both of which have interventricular communications and a hypoplastic left ventricle, are arguably not eligible to be categorized as having classical HLHS because they do not meet the traditional definition of having intact ventricular septum or intact atrioventricular septum. In addition to their hypoplastic left ventricles, if these hearts had one or more of the expected left-sided obstructive features seen in classical HLHS, such as aortic stenosis/atresia or mitral valve atresia/stenosis, hypoplastic aorta, or a coarctation of the aorta, they have been described as HLHS variants. The classification of these heart types remains unsettled; therefore, HLHS variants should be classified based not only on morphology but also on genetic studies, rather than on the need for single-ventricle surgical palliation or on clinical similarities to true HLHS. Regardless, these HLHS variants still require single-ventricle surgical palliation, irrespective of aorta size (2, 15).

**Figure 6:**
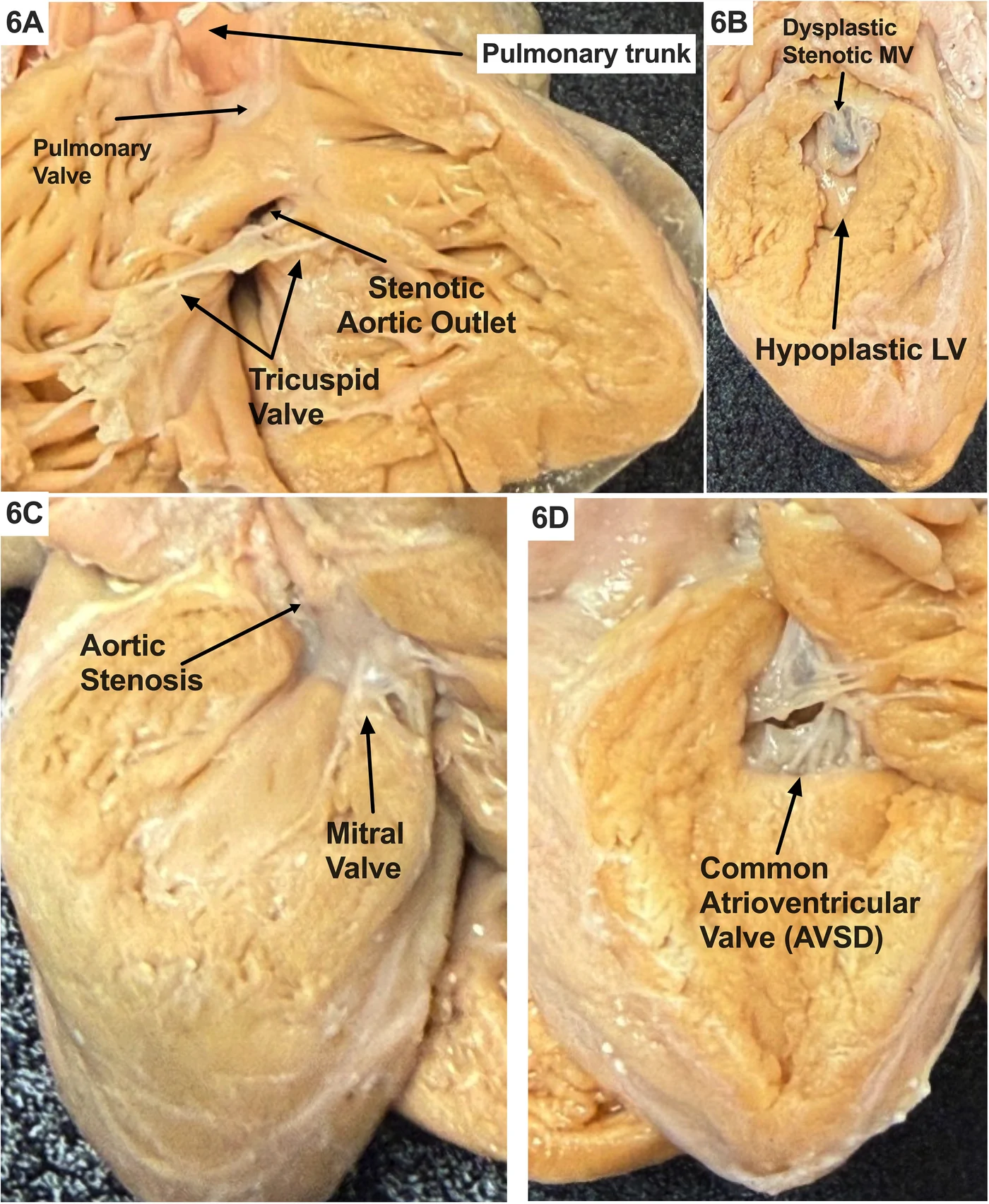
Specimens with hypoplastic left ventricles that do not meet the criteria of classical HLHS. **(A)** Right ventricle view of a heart with DORV and subaortic outflow stenosis, and a ventricular septal defect (not in image), **(B)** left ventricular view of A showing the hypoplastic left ventricle and the stenotic, dysplastic mitral valve; **(C)** aortic stenosis, hypoplastic left ventricle, and intact ventricular septum; **(D)** left ventricular view of an unbalanced atrioventricular septal defect (AVSD) with a dominant right ventricle and a hypoplastic left ventricle (LV).

## 3 Section II: clinical management

### 3.1 Early Detection

Since HLHS is now primarily diagnosed prenatally through fetal ultrasound, this early detection can inspire clinicians to pursue timely interventions like in-utero aortic valvuloplasty, offering hope for improved outcomes (16). Early detection of HLHS through fetal ultrasound is crucial, as it directly influences management strategies and intervention timing.

### 3.2 Pre-operative care of HLHS

The management of patients with HLHS with competing parallel circulation is challenging. Preoperative management of HLHS requires knowledge of the fetal circulation. In patients with aortic atresia and very severe aortic stenosis, there is a lower pO2 to the fetal brain, with probable decreased blood flow to the brain as well. The coronary flow is retrograded from the diminutive ascending aorta. Normally, the lungs receive only 11% of the combined ventricular output (17). In the normal fetus, oxygenated blood returning from the placenta enters the right atrium; it crosses the foramen ovale or an atrial septal defect to fill the left atrium. If the atrial communication is restricted or the foramen ovale is prematurely closed in the fetus with HLHS, blood becomes trapped in the left atrium, resulting in left atrial hypertension and secondary pulmonary venous congestion. The combination of chronic back-pressure injuries and remodeling of the developing pulmonary vascular bed, with altered mixing patterns, results in reduced oxygen tension and predisposes to pulmonary lymphangiectasis (19).

Postnatally, fully saturated pulmonary venous return must be routed to the right atrium due to the left ventricular inflow obstruction. The fully saturated pulmonary venous blood mixes with desaturated blood in the right atrium and is ejected by the right ventricle. By default, the right ventricle must supply both systemic and pulmonary circulations. Descending aortic blood flows retrograde through the PDA to supply the head and neck vessels and coronary arteries; therefore, brain and coronary ischemia can occur.

The balance between systemic vascular resistance and pulmonary vascular resistance plays a significant role in managing single-ventricle physiology. There is an inherent tendency for high pulmonary blood flow in otherwise uncomplicated cases, with concurrent systemic steal. The right ventricle is volume-overloaded at baseline. Therefore, the goals of pre-op care are to preserve ductal patency and balance pulmonary blood flow (Qp) and systemic blood flow (Qs). Maneuvers in the Pediatric Cardiac Intensive Care Unit to optimize Qp:Qs to 1 are challenging because of the competing parallel circulations.

### 3.3 Keeping the PDA open

The ductus arteriosus can be kept patent using prostaglandin (PGE1) or a PDA stent. The previously described starting dose range of 0.05–0.10 mcg/kg/min (20) has fallen out of favor, with a lower starting dose of 0.01 mcg/kg/min (21) now preferred. These previously higher doses were most likely the initial doses for patients who were not prenatally diagnosed and came to medical attention later in the neonatal period with a restrictive or closed ductus arteriosus. The risk of precipitating apnea is higher at high starting doses. The recommended maintenance dose of PGE is 0.01–0.04 mcg/kg/min. At our institution, we use doses as low as 0.003–0.005 mcg/kg/min while patients await surgical repair with a restrictive or closed PDA. Some institutions still maintain their patients on PGE for prolonged periods (2).

### 3.4 Approach to pulmonary overcirculation in the pre-operative stage

Preoperatively, flow to the systemic versus pulmonary vascular beds depends on their relative resistance. Clinicians also need to diagnose a high Qp:Qs state; some of the clinical manifestations of low cardiac output status include hypotension, decreased urine output, delayed capillary refill, and lactic acidosis. Patients may also present with tachypnea, increased work of breathing, and respiratory distress (2). Systemic vascular resistance is less amenable to independent pharmacologic manipulation than pulmonary vascular resistance. Therefore, pulmonary vascular resistance has become the target for balancing blood distribution (Qp: Qs) in patients with HLHS. Several medical or surgical treatments aim to prevent pulmonary overcirculation by manipulating pulmonary vascular resistance to improve systemic perfusion in parallel circulation. These temporary treatments allow patients with HLHS to reach maturity for surgical palliation. One method is sub-ambient oxygen, also known as a hypoxemic mixture, consisting of an inspired fraction of oxygen (FiO2) between 15% and 20% (22). Hypoxia can be introduced either by inhaled nitrogen (N2) or by hypercarbia using CO2 administration to diminish pulmonary blood flow (23–25). Tabutt et al. conducted a study comparing inhaled N2 and CO2 and found that both strategies induced the desired fall in Qp in humans. In their research, N2 therapy did not improve oxygen delivery (DO2), but CO2 therapy increased DO2 by 44% (22). However, the literature on hypoxic therapy in single ventricle patients is sparse for randomized controlled study. It is important to recognize that hypoxic therapy is a temporary measure and not a substitute for definitive surgical palliation. Careful monitoring of PaO2 levels is essential to ensure safety and effectiveness, which should instill confidence in the approach. Hesitation to use this widely stems from concerns about the cellular effects of a hypoxic mixture on tissue oxygenation. Another problem is that a non-intubated patient receiving a hypoxic mixture may develop hypoventilation and apnea when combined with prostaglandin administration; therefore, PaO2 levels will fall drastically (26). Using a hypoxemic mixture requires vigilance, with frequent blood gas checks to monitor PaO2 and base deficit. With decreased PaO2, the gas mechanics operate on the nearly vertical portion of the oxygen-hemoglobin dissociation curve; hence, a slight change in PaO2 results in a significant fall in SaO2 in patients with HLHS (23). To maintain Qp: Qs of approximately 1:1, the ideal PaO2 would be 35-45 mmHg. A caveat is that a central line adequately placed in the right atrium of a patient with HLHS and aortic atresia and/or very severe aortic stenosis is generally quite representative of arterial gas in such patients. A hypoxemic mixture can be administered via a high-flow nasal cannula, an oxy-hood, or a combination of both, and ultimately through the endotracheal tube of an intubated patient. Adjusting the ventilator of an intubated patient to allow for higher PaCO2 will also restrict pulmonary blood flow and blend CO2 into the circuit (24, 25). Although controversial, inhaled hypoxic therapy is one of the strategies to temporize a HLHS patient in a situation of excessive tachypnea and at risk of hemodynamic decompensation while waiting for surgical repair. The hypoxic mixture therapy cannot replace surgical palliation (23, 27).

### 3.5 Optimization of systemic circulation

One can increase systemic circulation by using afterload-reduction agents such as milrinone, which can be titrated to effect as we monitor saturations, clinical cardiac output, base deficit, lactate levels, and PaO2 (1). Optimization of oxygen-carrying capacity by keeping hematocrit > 40% is also ideal. Ultimately, if all these maneuvers fail and one still needs to wait for intervention, muscle relaxation may be necessary to take over respiratory support, fully manipulate PaCO2, and decrease metabolic demands.

### 3.6 Special considerations

#### 3.6.1 HLHS with intact or restrictive atrial septum

Neonates with a restrictive atrial septum account for only 6% of patients with HLHS, but are the most challenging subgroup to manage, with mortality rates of more than 50% (26). Key management before opening the atrial septum, whether through surgical intervention or cardiac catheterization, includes intubation, optimized ventilation, maintenance of adequate blood volume, correction of metabolic acidosis, inotropic support for low cardiac output, and PGE1 therapy. Increasing PGE may worsen the clinical picture, as it may transiently increase blood returning to the left atrium without egress. Administration of sodium bicarbonate to mitigate the acidotic milieu and alerting the cardiac catheterization and surgical teams are crucial. Atrial-level communication in a patient with HLHS is often superiorly positioned and very thick. As such, cardiac catheterization to establish atrial-level patency can be challenging. If a balloon atrial septostomy cannot be performed effectively, static ballooning may be attempted; if that fails, surgical atrial septostomy is indicated. The original form of this procedure is called the Blalock-Hanlon procedure (published in 1948 by Alfred Blalock and C. Rollins Hanlon) (29).

#### 3.6.2 HLHS with tricuspid regurgitation or poor right ventricular function

Infants with moderate to severe tricuspid valve insufficiency and/or poor right ventricular function from the very beginning pose an exceptionally high degree of challenge for the cardiac intensive care physician. In addition to critical care management of ventilation, diuretic therapy, and inotropic support, heart failure/cardiac transplant evaluation, and early supportive care/palliative care team involvement should be considered.

#### 3.6.3 Pulmonary vein obstruction

Patients with HLHS and pulmonary vein obstruction will develop marked hypoxemia over time. This hypoxemia may develop after a period of apparent wellness. These patients may need intubation, hyperventilation, increased FiO2, and increased systemic vascular resistance by using inotropic support to stabilize patients till surgical intervention can be performed. Depending on the severity of pulmonary vein obstruction and low cardiac output syndrome, the obstructed pulmonary venous return is a surgical emergency and may require rescue via extracorporeal membrane oxygenation (ECMO).

Single-ventricle patients born with normal pulmonary venous connections but who developed pulmonary vein obstruction after initial surgical intervention carry a high risk of failing to achieve Fontan completion (30). This obstruction is secondary to external compression with cardiomegaly, especially on the left side.

### 3.7 Section IIB: surgical intervention

Dorty and Knott reported the first attempt at surgical palliation for HLHS in 1977 (31). All patients died of right ventricular failure or coronary ischemia. Subsequently, Norwood and colleagues described the first successful palliative operation, which remains the foundation of current management (32). The standard surgical strategy for palliation of patients with HLHS consists of three stages: the Norwood procedure, the bidirectional Glenn shunt, and the Fontan procedure (33). This staged approach aims to progressively transform physiology from a parallel, pulmonary- and ductal-dependent systemic circulation to a more efficient, in-series circulation, in which systemic venous return is completely and directly channeled to the pulmonary arteries and the functional ventricle is solely responsible for the systemic circulation.

Each stage has a well-defined physiological goal, achieved through targeted surgical modifications of cardiovascular anatomy.

#### 3.7.1 Stage 1—the Norwood procedure

The physiological objectives are 1) to reconstruct an outflow tract capable of ejecting blood effectively into the systemic and coronary circulations by amalgamating the aortic and pulmonary roots (Damus-Kaye-Stansel procedure); 2) to create an unobstructed pathway to the systemic circulation by way of extensive neo-aortic reconstruction; 3) to establish a reliable source of pulmonary blood flow—either through a systemic-to-pulmonary artery shunt (modified Blalock–Taussig-Thomas) (Figures 7A,C) or a right ventricle-to-pulmonary artery (Sano modification) shunt (Figures 7B,D); and 4) to create an adequate site for complete atrial mixing that allows oxygenated pulmonary venous blood to reach the systemic ventricle by performing an atrial septectomy (34).

**Figure 7:**
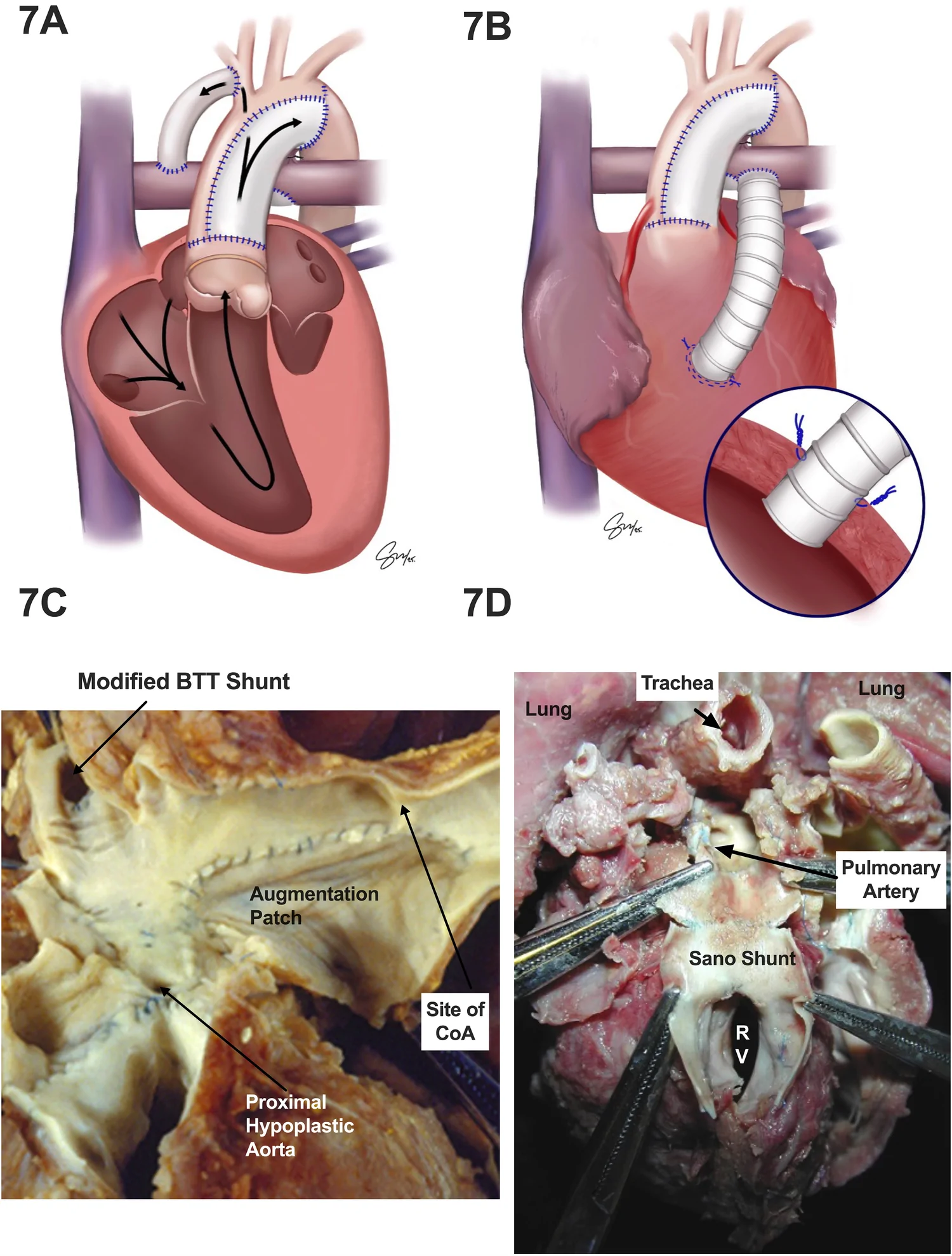
**Surgical palliation for HLHS: Norwood procedure**. (**A**) Norwood procedure with modified BTT shunt, (**B**) Norwood procedure with a Sano shunt. (Graphic illustration by Dr. Carlos Eduardo Diaz-Castrillon) (**C**) Pathologic specimen with Norwood procedure, an open Norwood arterial trunk, and a modified BTT shunt. (**D**) Pathologic specimen with Norwood procedure, an open right ventricle-to-pulmonary artery conduit (Sano Shunt). BTT: Blalock-Taussig-Thomas shunt. CoA: coarctation of the aorta.

#### 3.7.2 Cannulation and perfusion strategies

Arterial cannulation may be performed through the main pulmonary artery or innominate artery, or through dual cannulation of the innominate artery and descending aorta, using both the innominate and descending aorta, to maintain continuous perfusion of all vascular beds under mild hypothermia (35, 36). During cooling, a pH-stat strategy may confer advantages in cerebral protection and postoperative recovery by increasing cerebral flow and oxygenation, improving homogeneous cooling, and reducing ischemia–reperfusion injury in neonatal cardiac surgery with deep hypothermia (37).

Early neonatal arch repairs were traditionally performed under deep hypothermic circulatory arrest (DHCA) to provide a bloodless operative field and optimal surgical exposure; however, this strategy entails a period of global cerebral ischemia followed by reperfusion injury. Subsequent techniques incorporating low-flow cardiopulmonary bypass (CPB) for cerebral perfusion sought to maintain partial brain perfusion, thereby reducing ischemic insult (38). Prolonged DHCA is linked to worse neurodevelopmental outcomes (39). In a landmark trial from Boston Children's Hospital, infants undergoing circulatory arrest had lower psychomotor scores at one year compared with those receiving low-flow bypass, with deficits evident beyond 35–45 min of DHCA (40). Subsequent follow-up demonstrated early motor, language, and behavioral impairments and later deficits in visual-spatial and executive functions, which are largely attenuated by adolescence (41–43).

Selective antegrade cerebral perfusion (SACP) has become the preferred cerebral protection strategy during neonatal aortic arch reconstruction, replacing DHCA in many centers. Although practices vary, cold cardioplegia solution is first administered through the arterial cannula to protect the myocardium. A brief period of DHCA is often used to perform the atrial septectomy while the heart is exsanguinated. This is then followed by initiation of SACP for arch reconstruction, with resumption of complete cardiopulmonary bypass once the patch anastomosis to the amalgamated neo-aorta has been completed. Perfusion is then resumed at low flow rates (commonly 10–20 mL/kg/min), while arterial pressure and cerebral oxygen saturation are closely monitored to ensure adequate cerebral protection (44). Over time, the target temperature for SACP increased to moderate hypothermia (22–28 °C), and continuous low-flow cerebral perfusion became more common to minimize ischemic injury (44, 45).

Randomized trials comparing SACP and DHCA demonstrated no significant differences in survival or neurodevelopmental outcomes. However, patterns of brain injury differed—central lesions were more common with SACP and peripheral lesions with DHCA (46). Experimental models indicate that DHCA induces greater neuronal apoptosis and metabolic disruption than SACP at equivalent temperatures (47).

However, SACP strategy varies significantly across institutions. In a comprehensive review of 57 studies published between 2000 and 2023, Catalano et al. found wide heterogeneity in flow rates (10–100 mL/kg/min), target temperatures (18–34 °C), and monitoring practices, including inconsistent use of near-infrared spectroscopy (NIRS), electroencephalography (EEG), and transcranial Doppler (48).

#### 3.7.3 Sustained total all-region (STAR) perfusion

More recently, perfusion strategies have evolved beyond regional protection to maintain continuous whole-body perfusion throughout Norwood reconstruction. One such technique, Sustained Total All-Region (STAR) perfusion, developed at Duke University, eliminates ischemic intervals to the brain, myocardium, and lower body while allowing repair under mild hypothermia (32–34 °C) (49). STAR perfusion employs simultaneous cannulation of the innominate artery, descending aorta, and aortic root, ensuring continuous antegrade cerebral, coronary, and systemic flow. In a series of 80 infants (2012–2020), STAR perfusion significantly reduced CPB and operative times (171 vs. 245 min; 331 vs. 502 min, *p* < 0.0001), lowered peak lactate (8.1 vs. 9.9 mmol/L), and accelerated lactate normalization (18 vs. 27 h). Survival to discharge was higher (97% vs. 85%), with lower mortality (2.7% vs. 10.3%) and no seizures (0% vs. 10.9%) compared with standard SACP Norwood repairs. These results suggest improved metabolic stability, reduced coagulopathy, and faster recovery, though larger multicenter studies are needed to confirm long-term benefits (50).

#### 3.7.4 Aortic arch reconstruction

This step begins with extensive dissection of the aortic arch, head and neck vessels, and proximal descending thoracic aorta, with particular care taken to avoid injury to the recurrent laryngeal nerve. Most centers excise the aortic isthmus and adjacent intercostal arteries to ensure full removal of ductal tissue (Figure 8A). In contrast, our approach preserves the posterior wall at the aortic isthmus to maintain anatomic continuity (Figure 8B). The incision extends from the proximal descending thoracic aorta along the inferior arch margin toward the ascending aorta, ending just above the pulmonary root, which is typically divided from the pulmonary artery confluence before the arch reconstruction.

**Figure 8:**
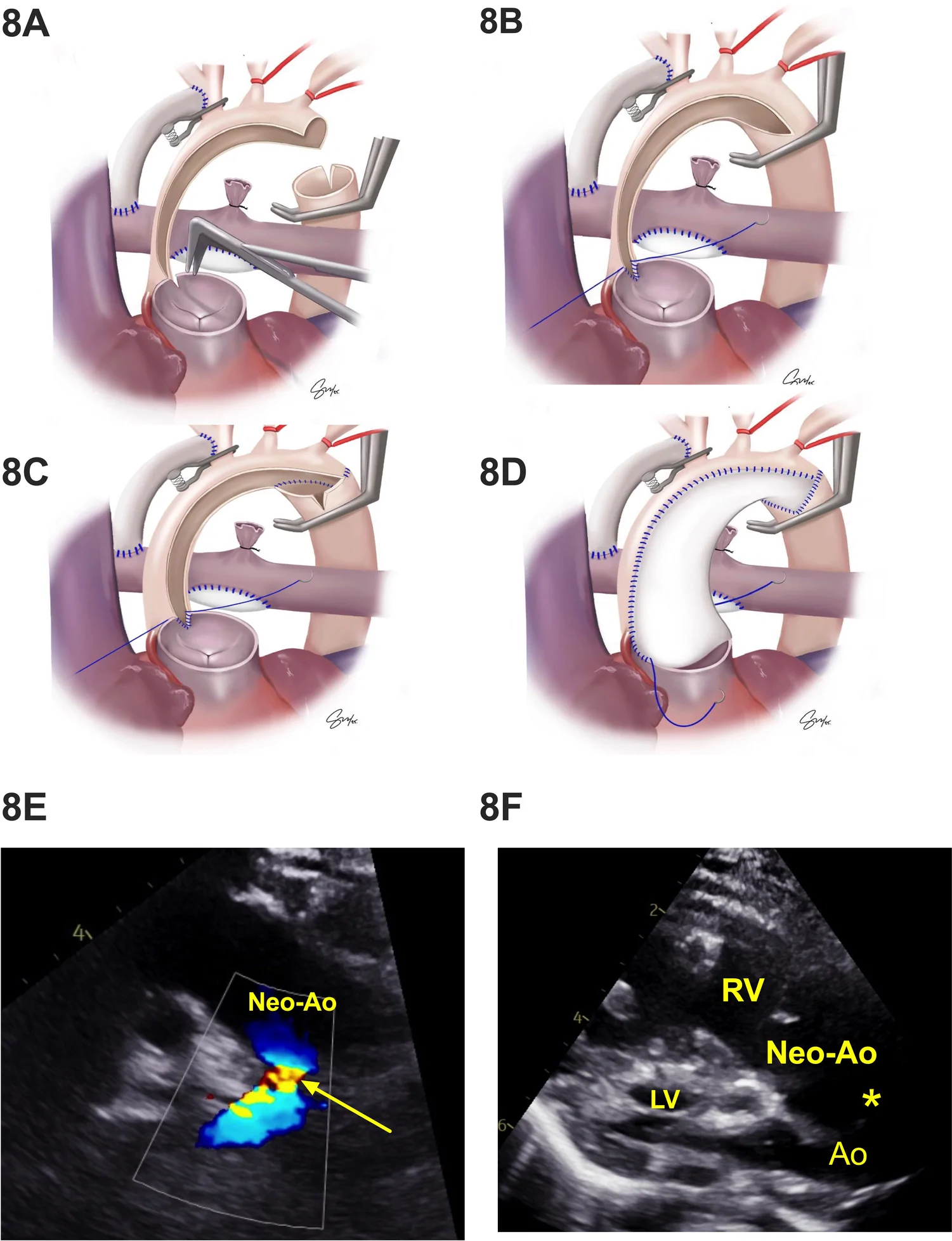
Surgical palliation for HLHS: Norwood procedure. **(A–D)**: ascending aorta and aortic arch reconstruction with the Damus–Kaye–Stansel procedure. Graphic illustration by Dr. Carlos Eduardo Diaz-Castrillon. **(E)** TTE suprasternal notch view of the aortic arch following reconstruction with residual coarctation (arrow); **(F)** TTE parasternal long-axis view showing hypoplastic native aortic root (Ao) communicating with the neo-aortic root (Neo-Ao) via a Damus–Kaye–Stansel anastomosis (*) following the Norwood procedure. LV: left ventricle, RV: right ventricle.

A Damus–Kaye–Stansel (DKS) anastomosis is then performed to connect the divided main pulmonary artery with the hypoplastic ascending aorta, creating a unified outflow tract (Figures 8C,D). Different techniques exist for constructing the neo-aortic root (Figure 8F), but the goal remains to establish a wide, non-stenotic channel that preserves coronary flow and ensures effective systemic circulation (Figures 8D,F).

The isthmus and transverse arch are subsequently augmented, usually with a decellularized pulmonary homograft patch. When the isthmus and ductal tissue are completely excised, the distal anastomosis is performed using an interdigitating technique incorporating a posterior–lateral incision to ensure optimal alignment. In our technique, posterior interdigitation is unnecessary; instead, an anterior longitudinal incision in the descending aorta allows placement of an anterior patch, achieving a wide, tension-free reconstruction while preserving the native posterior wall. The patch is trimmed and fashioned during anastomosis to create a gradual taper between the neo-ascending aorta reconstructed through the DKS anastomosis and the proximal descending thoracic aorta. This taper decreases the risk of branch pulmonary artery and bronchial compression. The Norwood arch reconstruction is completed once the patch is sewn to the borders of the neo-aorta (Figures 8A–D).

#### 3.7.5 Systemic-to-pulmonary shunt

The systemic-to-pulmonary shunt is a key component of the Norwood procedure, providing pulmonary blood flow, and the choice among options significantly influences patient prognosis and surgical success.

- Modified Blalock–Taussig–Thomas shunt (MBTT): A systemic-to-pulmonary connection made of expanded polytetrafluoroethylene (ePTFE, Gore-Tex) (Figures 7A,C) connecting the right or left subclavian artery (or brachiocephalic trunk) to the ipsilateral pulmonary artery. Tube diameter usually ranges from 3.5 mm (for neonates 2.5–3.5 kg) to 4 mm for larger infants. Oversized shunts may cause pulmonary over-circulation and decreased systemic perfusion (“systemic steal” or “diastolic runoff”). In contrast, undersized or overly long shunts may lead to pulmonary hypoperfusion and hypoxemia, as well as branch pulmonary artery stenosis due to distortion.
- Right ventricle–to–pulmonary artery shunt (Sano shunt) (Figures 7B,D): Constructed from PTFE, this conduit connects the right ventricle directly to the pulmonary artery or main pulmonary trunk (Figures 7B,D). The typical diameter is 5 mm, though 6 mm may be used when elevated pulmonary vascular resistance is anticipated. The Sano shunt provides pulsatile flow dependent on ventricular function and has lower flow resistance than the mBTTS. Similar to the mBTTS, improper estimation of the Sano shunt length can distort the branch pulmonary artery. The Sano shunt can be routed to the pulmonary artery confluence either to the left or right of the neo-aorta.

Several technical modifications have been developed to optimize outcomes and reduce complications such as conduit stenosis. These modifications include transmural insertion (51), the use of ring-reinforced conduits (52), and the incorporation of a valve (52) within the graft to reduce diastolic runoff and improve early ventricular and end-organ recovery (53). Collectively, these refinements aim to mitigate proximal conduit stenosis, excessive diastolic runoff, and ventricular dysfunction, and have been associated with improved short-term outcomes and reduced need for early reintervention.

In the landmark Single Ventricle Reconstruction trial, transplant-free survival was higher with the Sano shunt at 1 year (74% vs. 64%, *P* = 0.01), but not at 6 years (64% vs. 59%, *P* = 0.25) or 12 years (59% vs. 54%, *P* = 0.11); right ventricular ejection fraction (RVEF) also remained similar (51% vs. 52%, *P* = 0.43). The Sano group, however, showed higher rates of protein-losing enteropathy (5% vs. 2%, *P* = 0.04) and more catheter-based interventions (14 vs. 10 per 100 patient-years, *P* = 0.01) (4, 34, 54). The higher 1-year transplant-free survival observed with the Sano shunt has contributed to its preference at many centers.

Despite technical refinements, the Norwood operation remains high risk. In the Single Ventricle Reconstruction cohort, predictors of mortality included low birth weight, genetic anomalies, ECMO use, open sternotomy, and prolonged CPB or arrest times (55). An STS analysis of 2,557 patients reported 22% hospital mortality, rising from 17% with one complication to 45% with five or more complications (56). The most frequent complications were respiratory failure (21.6%), arrhythmias (19.2%), low cardiac output (16.2%), and cardiac arrest (12.2%). Rare but fatal events such as renal failure requiring dialysis (0.5%) carried a >90% mortality. Overall, low weight, genetic anomalies, and preoperative instability strongly correlated with poor outcomes (56). These data suggest that HLHS patients with multiple high risks may benefit from a less invasive hybrid approach.

#### 3.7.6 Stage 1—hybrid alternative

The physiological objectives are 1) to secure adequate systemic perfusion, 2) to maintain effective interatrial communication for blood mixing, and 3) to regulate pulmonary blood flow to avoid pulmonary over-circulation and to reduce volume load on the single systemic ventricle (57–60). Unlike the classic Norwood procedure, the hybrid approach avoids CPB and deep hypothermic exposure, thereby reducing the risk of neurologic and hemodynamic injury in the neonate. The hybrid strategy consists of three key components:

- *Pulmonary artery (PA) banding:* Clinical and computational models suggest that an internal band diameter of approximately 2 mm on each pulmonary branch achieves a Qp: Qs ratio near 1:1, optimizing mixed venous saturation and systemic oxygen delivery. The final band size, however, should be individualized based on patient weight and branch anatomy. A practical method is to calculate band length as body weight (kg) + 7 mm for the left pulmonary artery and weight + 7.5 mm for the right, using a 2-mm-wide tape on both branches to protect the lungs from sustained high pressures and flows. Alternatively, 3-mm-wide bands can be fashioned from a 3.5-mm ePTFE tube and progressively constricted. The degree of constriction should lower systemic oxygen saturation to 75%–85% and generate an intraoperative trans-band gradient of 30–50 mmHg. Pulmonary artery bands are generally placed before ductal stent implantation, as subsequent access to the left pulmonary artery becomes technically more challenging and may jeopardize stent stability. To reduce complications, the bands are positioned as proximally as possible to minimize distal migration and facilitate removal during the next surgical stage (Figure 9A).
- *Ductal stent implantation:* Introduced directly through a sheath placed in the main pulmonary artery, this approach avoids hemodynamic collapse secondary to acute tricuspid or pulmonary valve insufficiency. This complication can arise from distortion during traditional guidewire passage in hearts with HLHS. To optimize this technique, only a very short segment of the sheath should enter the main pulmonary artery, ensuring that the deployed stent fully covers the proximal ductal orifice and prevents stenosis (Figures 9B,C).
- *Balloon atrial septostomy* to ensure adequate oxygenated blood mixing.

**Figure 9:**
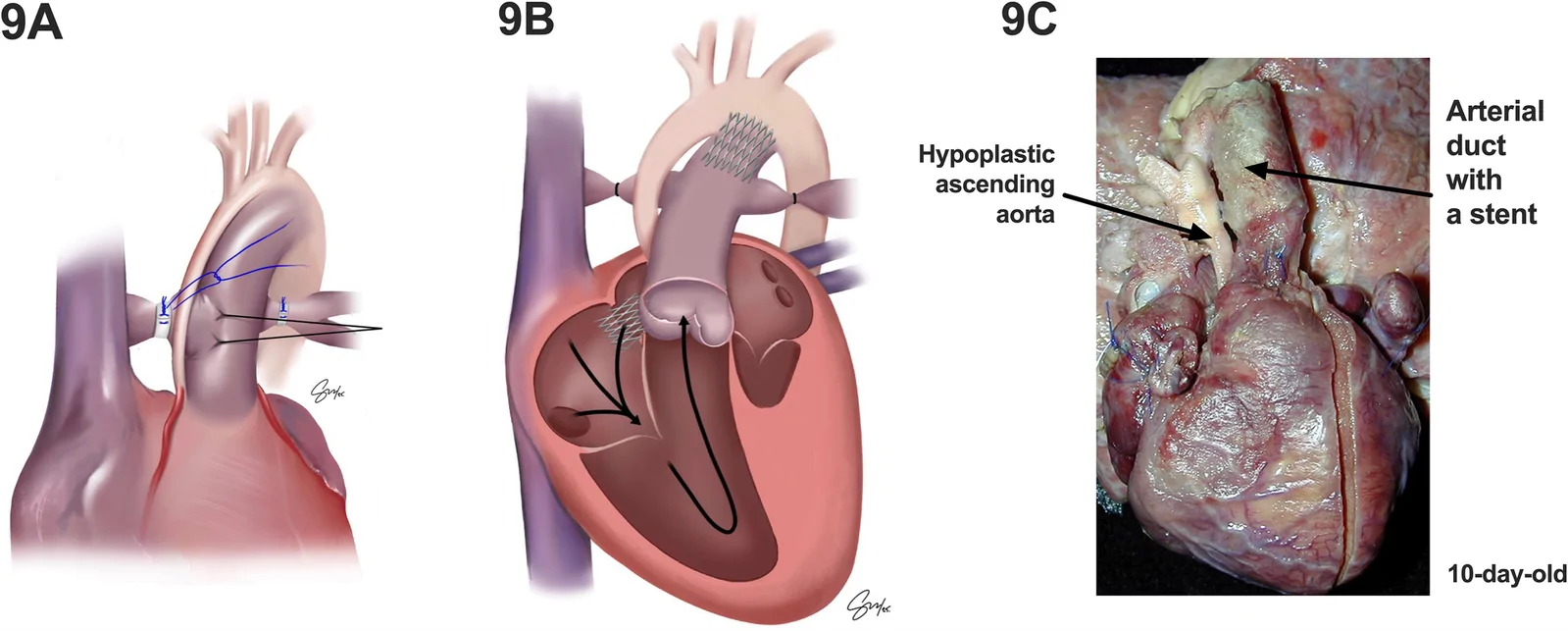
**Surgical palliation for HLHS: Hybrid procedure.** (**A**) Hybrid procedure with bilateral pulmonary artery band placement; (**B**) PDA stent and atrial stent. Graphic illustration by Dr. Carlos Eduardo Diaz-Castrillon. (**C**) Pathological specimen demonstrating a hybrid procedure with a PDA stent (arrow) and pulmonary bands (not shown).

Evidence regarding the hybrid strategy for Stage 1 palliation in HLHS supports its role as a valid alternative to the Norwood procedure, particularly in neonates deemed high-risk. First described in 1993 by Gibbs (57), the hybrid technique offers the possibility of postponing CPB-dependent reconstruction beyond the neonatal period (59). However, most centers reserve the hybrid approach for candidates at high postoperative mortality risk (prematurity, low birth weight, hemodynamic instability, or non-cardiac/neurological lesions) (61–63). It can also serve as an intermediate strategy for patients who may be suitable for later biventricular repair (64) or as a bridge to transplantation (65).

The hybrid approach is used in only a small proportion of centers (13%), typically in lower-volume programs with specific patient selection criteria such as high surgical risk or anatomical considerations, which may influence outcomes. Contemporary series showed that operative mortality rose sharply with risk category—1% for standard, 14% for intermediate-, and 27% for high-risk cases (*P* < 0.001) (66). Despite greater preoperative risk among hybrid candidates (low birth weight, prematurity, organ dysfunction), propensity-matched outcomes showed comparable hospital mortality between hybrid and Norwood procedures (19.1% vs. 19.4%; *P* = 0.95) (67). Collectively, these findings suggest that under equivalent risk conditions, the hybrid strategy achieves survival outcomes comparable to those of the conventional Hybrid palliation can also be performed in the cardiac catheterization laboratory using a fully transcatheter approach, with ductal stenting and pulmonary artery flow restrictors. Therefore, hybrid palliation enables high-risk patients to survive the volatile neonatal period, allowing a delayed Norwood procedure or a comprehensive stage 2 (Norwood/Glenn) procedure. Some centers are extending their use to lower-risk patients, but consensus on its routine adoption remains limited.

#### 3.7.7 Postoperative management of HLHS after first-stage palliation

Following the Norwood procedure, pulmonary blood flow is supplied through either a modified Blalock-Taussig-Thomas or Sano shunt. Initial management of patients with a Norwood/MBTT shunt again still requires balancing Qp:Qs. Taeed et al. demonstrated that pulmonary venous desaturation is common after the Norwood procedure, and that SaO2 alone is not sufficient to predict Qp and Qs after the Norwood procedure (68). Judiciously optimizing pulmonary venous oxygenation by using inspired oxygen and adjusting positive end-expiratory pressure (PEEP) is critical in postoperative care. If the shunt is relatively large, excessive pulmonary blood flow may occur. In such cases, the previously described maneuvers can be implemented to balance pulmonary and systemic blood flow. However, if the shunt is long and relatively resistive, the patient has high pulmonary vascular resistance and will require increased systemic vascular resistance to drive flow through the shunt, using increased volume, epinephrine, norepinephrine, vasopressin, and calcium. In extreme cases, pulmonary vasodilators, such as inhaled nitric oxide (iNO), are used to balance Qp:Qs. These maneuvers are intended to be temporary as the infant's circulation adapts. Heparin or cangrelor (69) helps ensure shunt patency in the immediate postoperative period. Anticoagulation could later be transitioned to enoxaparin, aspirin, or both. Aspirin was noted to lower the risk of shunt thrombosis in a multicenter, observational study (70). VerifyNow Aspirin Reactivity Unit (ARU) monitoring is reported to reduce the need for escalation of care in patients with congenital heart disease requiring systemic-to-pulmonary artery shunts (71). Clopidogrel therapy with or without aspirin in infants with a systemic-to-pulmonary-artery shunt did not reduce either shunt-related morbidity or mortality (72). The anticoagulation regimen during the interstage and after Fontan completion is institution-dependent (73).

Management after a hybrid procedure can be similar to that after a Norwood/M BTT shunt; balancing Qp: Qs is important, as is maintaining ductal patency with anticoagulation and/or antiplatelet therapies. Closely monitor patients for signs of stent migration into the pulmonary artery or aorta, as well as PA band migration, to prevent hemodynamic compromise, and screen for retrograde arch obstruction due to stent migration with daily 4-limb blood pressure measurements. Interval echocardiographic assessment can assist in early diagnosis, enabling timely intervention and optimizing patient outcomes (2).

### 3.8 Glenn shunt

#### 3.8.1 Stage 2: bidirectional superior cavopulmonary connection (Glenn procedure)

The physiological objective is to partially divert systemic venous return from the right atrium by allowing passive flow of desaturated blood from the superior vena cava (SVC) directly into the pulmonary circulation. This marks a key transition toward the in-series Fontan circulation, substantially unloading the single ventricle and helping preserve mid-term ventricular function. The source of pulmonary blood flow also transitions from a fixed-diameter surgical shunt to the superior vena cava, allowing the potential for growth with the patient (Figures 10A,B).

Bidirectional cavopulmonary anastomosis and takedown of the systemic-to-pulmonary shunt: The previously placed MBTT or Sano shunt from the Norwood stage is dissected, divided, and ligated. Afterward, the SVC is divided and anastomosed to the right, left, or both pulmonary arteries, depending on patient anatomy. The azygos vein is typically ligated and divided to avoid decompression of the Glenn circulation. When a size mismatch exists between the SVC and the pulmonary artery—or when either is hypoplastic—a patch may be required to enlarge the anastomosis, thereby preventing pressure gradients and reducing the risk of stenosis. At our institution, we routinely use a PTFE patch to augment the anterior aspect of the anastomosis, extending it toward the confluence of the pulmonary branches (Figures 10A,B). Other commonly used materials include autologous or bovine pericardium. The anastomosis should provide an unobstructed pathway from the SVC to both pulmonary arteries, optimizing bilateral pulmonary blood flow and minimizing the risk of thrombosis.

**Figure 10:**
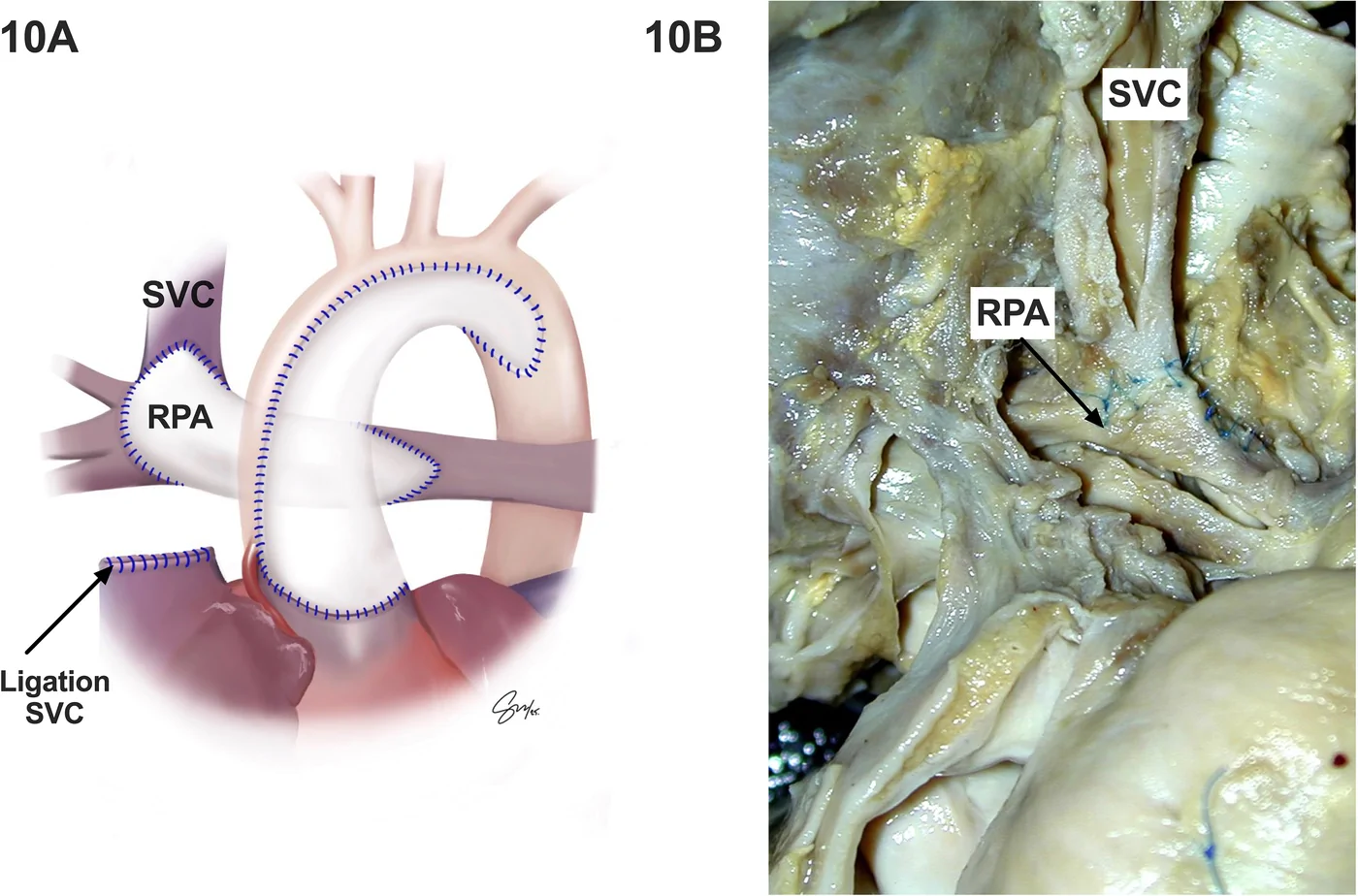
**Surgical palliation for HLHS: Glenn procedure.** **(A)** graphic illustration by Dr. Carlos Eduardo Diaz-Castrillon. **(B)** The pathologic specimen demonstrating a bidirectional Glenn procedure.

This stage markedly reduces ventricular volume load and improves systemic oxygenation by increasing pulmonary blood flow, although complete normalization of saturation is uncommon. It also promotes pulmonary artery growth, a key determinant of Fontan success. Reported mortality after Stage 2 palliation ranges from 5% to 10% in contemporary multicenter series, with lower rates at high-volume centers (74). The downside of Stage 2 palliation is the development of venovenous collateral circulation due to elevated pressure in the Glenn connection, leading to reduced pulmonary blood flow and increased cyanosis; this situation can promote the development of aortopulmonary collaterals, which offset the ventricular unloading provided by the Glenn, potentially resulting in reduced systemic ventricular function. Major complications include renal failure, ECMO support, ventricular dysfunction, arrhythmias, and unplanned reinterventions. In the STS analysis by Baker-Smith et al. (1,862 patients, 2007–2012), hospital mortality was 4.5% overall, rising to 11% with one major complication and 57% with three or more. Mortality correlated most strongly with ECMO (OR 30.5), renal failure requiring dialysis (OR 8.6), cardiac arrest (OR 6.8), and unplanned reoperation (OR 3.5) (75).

Performing Glenn around 3 months of age, as supported by CHSS data, underscores the importance of timing to optimize survival and foster confidence in clinical decision-making (76). Institutional experience at the Children's Hospital of Pittsburgh supports this window, reporting a median age of 100 days (IQR 87–119), routine use of ePTFE patch augmentation in 90% of cases, no operative deaths, and comparable late survival (77). In stable patients with adequate pulmonary artery growth and preserved ventricular function, the procedure may be advanced slightly before 90 days, provided that individualized assessment and close interstage monitoring are in place.

#### 3.8.2 Postoperative management after second-stage palliation

Postoperative management after the Glenn procedure is generally less challenging than after first-stage palliation. Selected patients with favorable preoperative hemodynamics may undergo early extubation, including in the operating room.

Postoperative care focuses on avoiding high PEEP and high pulmonary vascular resistance states to optimize flow in the Glenn circulation. By avoiding hyperventilation, one can manipulate PaCO2 in an intubated patient. Often, a combination of milrinone and epinephrine drips postoperatively is necessary to augment cardiac inotropy. Cerebral congestion from the new passive circulation to the pulmonary arteries can cause significant patient discomfort. Adequate pain control and simply elevating the head of the bed can significantly improve patient comfort by using gravity to promote anterograde blood flow from the cerebral to the pulmonary circulation (2).

Monitoring these patients for desaturation relative to their physiology and assessing the common causes is crucial. The differential diagnosis of a desaturated Glenn patient includes:

1. Pulmonary venous desaturation, which may be managed with supplemental oxygen and interventions to improve lung aeration, including management of conditions such as atelectasis, pneumothorax, and hemothorax. These patients require monitoring for high chest tube output and pleural effusions/chylothorax due to changes in systemic venous pressure, injury to the thoracic duct or lymphatic system during surgery, central lymphatic flow disorder, or pulmonary lymphatic perfusion syndrome (78).
2. Inadequate pulmonary blood flow, which can be treated with inhaled nitric oxide to decrease pulmonary vascular resistance. Cardiac catheterization should be performed to investigate possible causes. If the patient requires additional pulmonary blood flow, an additional systemic-to-pulmonary artery shunt may be considered to augment pulmonary blood flow (79).
3. Systemic desaturation, such as sepsis or anemia; therefore, ensuring an adequate hemoglobin level for Glenn physiology is important.

### 3.9 Fontan-Kreutzer completion

#### 3.9.1 Stage 3: total cavopulmonary connection (Fontan–Kreutzer or Fontan completion procedure)

The physiological objective is to complete the transition to the in-series Fontan circulation, in which systemic venous return is directed entirely into the pulmonary circulation without passing through the functional ventricle. This eliminates mixing of oxygenated and deoxygenated blood, improves arterial oxygenation, and further reduces single-ventricle volume load.

In the Fontan circulation, systemic venous blood passively traverses the pulmonary circulation, driven solely by the pressure gradient (trans-circulatory pressure) between the systemic veins and pulmonary arteries. Without a subpulmonary ventricle to propel flow, cardiac output becomes limited by pulmonary vascular resistance. Long-term Fontan success depends on maintaining low pulmonary vascular resistance, appropriate central venous pressures, and unobstructed pathways for systemic venous return.

The classical, direct atriopulmonary Fontan procedure was challenging to adapt to HLHS. It was fraught with the risk of sinus node dysfunction, atrial arrhythmias, right atrial dilation, and baffle obstruction when attempting to partition systemic and pulmonary venous blood. Thus, two principal approaches currently exist for Fontan completion:

- Lateral tunnel (LT): A baffle is constructed within the lateral aspect of the right atrium to redirect inferior vena cava (IVC) flows to the pulmonary arteries through the stump of the SVC disconnected during the Glenn procedure (Figure 11A). This method avoids the baffle obstruction seen with the classical Fontan and reduces the amount of right atrial tissue in the systemic venous portion of the circuit. However, atrial tissue remains exposed to elevated pressures, increasing the long-term risk of atrial tachyarrhythmias and potential baffle leaks. In addition, cardioplegic arrest is needed to sew the baffle.
- Extracardiac conduit (ECC): Introduced in the 1980s, this method connects the IVC directly to the pulmonary arteries using an external ePTFE conduit (Figures 11B–E). Under CPB, the IVC is disconnected from the right atrium, and an 18 mm or 20 mm ePTFE tube is sewn to connect the IVC to the underside of the ipsilateral branch pulmonary artery. Typically, the ECC is sited under the Glenn anastomosis to ensure equal distribution of IVC flow through the pulmonary arteries. The ECC avoids atrial incisions, reducing the risk of sinus node dysfunction and supraventricular arrhythmias, and can be used in complex anatomies (heterotaxy, anomalous venous drainage, etc.). Cardioplegic arrest is also not required.

**Figure 11:**
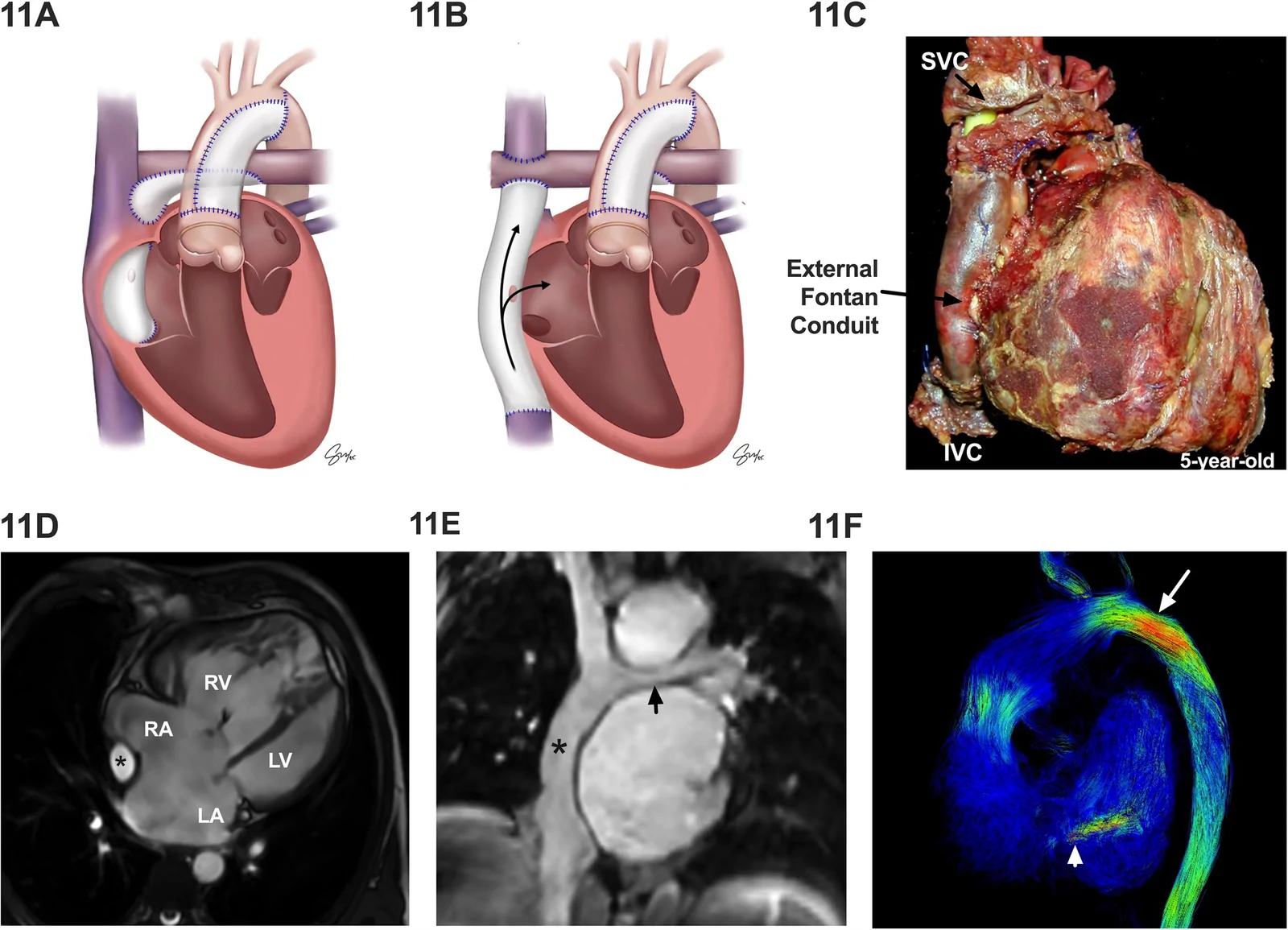
Surgical palliation for HLHS: Fontan completion: illustration of **(A)** lateral tunnel and **(B)** extracardiac Fontan completion. Graphic illustration by Dr. Carlos Eduardo Diaz-Castrillon. **(C)** Pathologic specimen demonstrating Fontan with an extra-cardiac conduit connecting IVC and SVC. **(D)** 4-chamber bSSFP cine MRI depicting hypoplastic left ventricle, dilated right ventricle, unrestrictive ASD, extracardiac Fontan conduit (*). **(E)** Coronal contrast-enhanced MR angiography showing a uniform-caliber Fontan conduit (*) with narrowing of the central left pulmonary artery (arrow) between the left atrium and dilated reconstructed ascending aorta; **(F)** sagittal view of 4D flow cardiac MRI rendered with velocity-encoded pathlines highlighting residual coarctation (arrow) and tricuspid valve regurgitation (arrowhead).

In some patients, a Fontan fenestration is performed intraoperatively by creating a small communication in the intra-atrial baffle (LT) or by forming a small anastomosis between the Fontan conduit and the right atrium (ECC). Highlighting the purpose of this procedure helps clinicians understand its role in surgical management and patient outcomes. The benefit of Fontan fenestration is to provide a small augmentation to systemic ventricular preload at the expense of a small degree of desaturation from the intentional right-to-left shunt. Fontan fenestration provides an alternative pathway for systemic venous return to augment ventricular preload and cardiac output, at the expense of systemic arterial desaturation due to intentional right-to-left shunting. There is a demonstrated benefit of decreased post-operative pleural effusion and, reportedly, reduced post-operative length of stay (80, 81). However, several studies suggest no long-term benefits to Fontan fenestration, with lower oxygen saturation and possibly increased risk of thromboembolic events (82, 83). The decision to fenestrate is surgeon-dependent; some fenestrate all Fontans, while others fenestrate selectively. These fenestrations either spontaneously close or can be closed with a transcatheter device to improve systemic saturations and exercise capacity (84). Routine transcatheter fenestration closure remains controversial.

Outcomes after the Fontan operation have improved markedly. Data from the combined EACTS–STS registry show that 65.1% of cases used an extracardiac conduit (ECC), 21.5% a lateral tunnel (LT), with a hospital mortality of just 2.3%. These advances should inspire confidence in progress of surgical techniques and patient care (85). Long-term results remain variable, but evidence suggests that performing the Fontan operation at an early age, ideally younger than 7 years, leads to more favorable outcomes. This emphasizes the importance of timely intervention for better patient prognosis and encourages proactive management (86). The optimal window for Fontan completion is generally considered to be between 2 and 4 years of age, with favorable midterm survival (87).

A meta-analysis of 19 studies (5,859 patients; mean follow-up 8.9 ± 2.6 years) reported 5-, 10-, and 15-year survival of 90.7%, 87.2%, and 87.5%, respectively (88). Another meta-analysis of 3,330 patients (1,729 ECC; 1,601 LT) showed superior 20-year survival with ECC (93% vs. 89%; *P* = 0.007) and greater arrhythmia-free survival (92% vs. 83% at 15 years; *P* < 0.0001) (89). Additionally, ECC was independently associated with reduced risk of systemic thromboembolic events (HR 0.20 vs. LT; 95% CI 0.04–0.97) (90).

Despite these benefits, late morbidity remains common. A meta-analysis of 61 ECC-only studies reported pooled incidences (per 100 person-years) of arrhythmia 1.8, thromboembolism 0.2, protein-losing enteropathy 0.7, conduit obstruction 0.7, reoperation 1.9, and late death 0.3 (91). Hepatic complications are also frequent: a systematic review of 5,701 patients (mean follow-up 17 years) found cirrhosis in 21% and advanced fibrosis in 30%, more often detected by imaging (26%) than biopsy (10%). Risk was correlated with older age at Fontan completion, ECC configuration, ventricular dysfunction, and elevated pulmonary capillary pressure (92). A study from the Australian and New Zealand Fontan registry (ANZFR) demonstrated that only 41% (95% CI: 33% to 51%) of patients who underwent Fontan completion were free of serious complications at 40 years of age (93). Complications after Fontan palliation are beyond the scope of this review; please refer to the review article on current treatment options for Fontan complications (94).

#### 3.9.2 Postoperative management after third-stage palliation

As in the Glenn procedure, acute postoperative management includes avoiding high PEEP and high pulmonary vascular resistance to optimize systemic venous return, with close monitoring for high chest tube output and pleural effusions or chylothorax to ensure prompt intervention (95, 96). In cases of acute Fontan failure, early consideration should be given to Fontan takedown (2, 97).

## 4 Section III Pathomechanisms of HLHS

### 4.1 Reduced blood flow – “no flow, no grow”

Reduced blood flow through the left side of the developing heart is commonly considered a likely etiology for hypoplasia of left-sided heart structures. This model proposes that abnormal blood flow adversely affects shear forces acting on the developing heart, leading to deficits in left ventricular growth. Experimental manipulation in chick embryos and fetal lambs to decrease left ventricular preload during embryonic development can result in cardiac malformations reminiscent of HLHS, such as obstructing left atrioventricular canal flow by inserting a balloon in the left atrium (98), or placing an occluder in the foramen ovale (99). Also observed in some cases of HLHS is restriction of the foramen ovale (100) or its premature closure (Figure 5). In addition to the hypothesis of reduced preload in the LV results in HLHS, a clinical study with longitudinal tracking of fetuses with aortic valve stenosis with a dilated or normally sized left ventricle in mid-gestation (16, 101) revealed left ventricular dysfunction, myocardial damage, and decreased left ventricular flow, with HLHS observed after birth (101, 102). However, aortic valvuloplasty in fetuses with aortic stenosis and evolving HLHS yielded little improvement in left ventricular growth (16), and single ventricle palliation remained necessary in most cases despite *in utero* aortic valvuloplasty. These findings suggest some HLHS patients may have intrinsic cardiomyocyte defects underlying their left ventricular hypoplasia, which has important implications for clinical management and targeted therapies. Longitudinal tracking of cardiac growth in mice with mutations causing HLHS revealed early LV hypoplasia, indicating intrinsic myocardial defects and supporting confidence in research progress (6).

### 4.2 Genetics of HLHS

HLHS has a high recurrence risk (103) and is associated with various chromosomal abnormalities (104, 105), findings that strongly support a genetic etiology of HLHS. Interestingly, first-degree relatives of probands with HLHS have a 10%-15% recurrence risk for a spectrum of left-sided cardiac lesions that include isolated bicuspid aortic valve (BAV) (103, 105–108) This suggests a complex genetic etiology of HLHS and helps explain the ongoing challenges in identifying its genetic causes. While multiple candidate genes and pathways have been identified, understanding their contribution to HLHS can inspire hope and curiosity about future therapies.

### 4.3 Genetic insights from human clinical studies

#### 4.3.1 Disorders associated with known syndromes and HLHS

There are various syndromic disorders in which HLHS may be observed. This suggests that genes associated with these syndromes may also play a role in HLHS, along with other genetic causes (109, 110). For example: Holt-Oram syndrome is caused by pathological variants in *TBX5* (109, 111)*,* Rubinstein-Taybi syndrome is caused by CREB-binding protein mutations (109, 112, 113); Smith–Lemli–Opitz syndrome results from recessive mutation in *DHCR7*, encoding 7-dehydrocholesterol reductase required for cholesterol synthesis (109), Noonan syndrome involves dominant mutations in *PTPN11* and other components of the RAS/MAPK signaling pathway (114, 115). Pathologic variants in chromatin modifier genes, including CHARGE association with CHD7 (116) variants and Kabuki syndrome, which is caused by dominant mutations in KMT2D or KDM6A, have been reported to be associated with HLHS cardiac phenotypes (117).

#### 4.3.2 Chromosomal abnormalities associated with HLHS

Chromosomal abnormalities were noted in 10% of HLHS cases, including trisomy 13 (118, 119), trisomy 18 (119, 120), trisomy 21 (119, 121), Turner syndrome (119, 122), and DiGeorge syndrome (119, 121). Notably, Turner syndrome with HLHS is associated with significant mortality (123, 124).

#### 4.3.3 Copy number variants

Copy number variants (CNVs) were associated with more than 10% of single ventricle lesions, including HLHS (125–127) with examples such as Jacobsen syndrome (11q24-qter deletion) (128), Alagille syndrome (20p12.2-p12.3 deletion) (129), dup 1q21.1, dup 16p13.11, dup 15q11.2-13, dup 22q11.2, and del 2q23.1 (130), del14q23.3 (131). However, copy number variants of undetermined significance in neonates with HLHS are not associated with worse surgical outcomes (127, 132).

We observed that a very common deletion CNV (delCNV) in the protocadherin alpha gene cluster is significantly associated with HLHS and BAV. BAV is a left-sided lesion often seen with HLHS, or as an isolated lesion in relatives of HLHS probands, suggesting an overlapping genetic etiology (133). Paradoxically, while HLHS is relatively rare, BAV is one of the most common birth defects, with a prevalence of 1–2% in the Caucasian population. While BAV is benign in early life, it is associated with late-onset morbidity/mortality due to aortic valve disease and/or aortic aneurysm. Such BAV-associated aortopathy accounts for >50% of adults undergoing valve surgery (134). This PCDHA delCNV is extremely common, seen in nearly 16% of Caucasians, less in African Americans, and even less in Asians (135), similar to the order of BAV prevalence in these respective populations, supporting a genetic linkage.

#### 4.3.4 Gene mutations

Given the genetic heterogeneity and likely oligogenic etiology of HLHS, identifying specific genes and their pathogenic variants causing CHD has been challenging. Nevertheless, pathogenic variants in several genes have been identified as significantly associated with HLHS. Examples of pathogenic variants associated with HLHS include *NOTCH1* (126, 136–138), *NKX2-5* (126, 137, 139), *HAND1* (126, 137, 140), *Celsr1* (141)*, ERBB4* (139), *Ets1* (143)*, GJA1* (144), *LRP2* (145), *Myh6* (146), *PROX1* (147), *PCDHA* deletion (135), *RBFOX2* (148, 149), *SMAD3* (150). Additionally, predicted pathogenic mutations in cardiomyopathy-associated genes—*MYBPC3, RYR2, and MYH6*—were noted in HLHS patients with cardiomyopathy, suggesting a shared clinical and genetic pathway between HLHS and cardiomyopathy (151).

### 4.4 Genetic architecture of HLHS- learning from the mouse models

Evidence of a genetic etiology for HLHS has also come from the recovery of 8 hypoplastic left ventricle (HLV) mutant mouse lines from a large-scale forward genetic screen (6) for mutations causing CHD. The HLV phenotype observed in these lines spanned a spectrum of phenotypic severity similar to what is observed clinically, i.e., comprising varying levels of LV, aorta, and aortic/mitral valve hypoplasia (Figure 12). Whole-exome sequencing analysis of the HLHS mutant mice showed no mutations/genes shared in common, indicating genetic heterogeneity. The observation of non-Mendelian transmission of HLHS in these 8 lines indicated complex genetics involving an oligogenic etiology for HLHS (6, 152). These findings suggest that the previous failure to recover HLHS mutants in mice likely reflects genetic heterogeneity and complex genetics.

**Figure 12:**
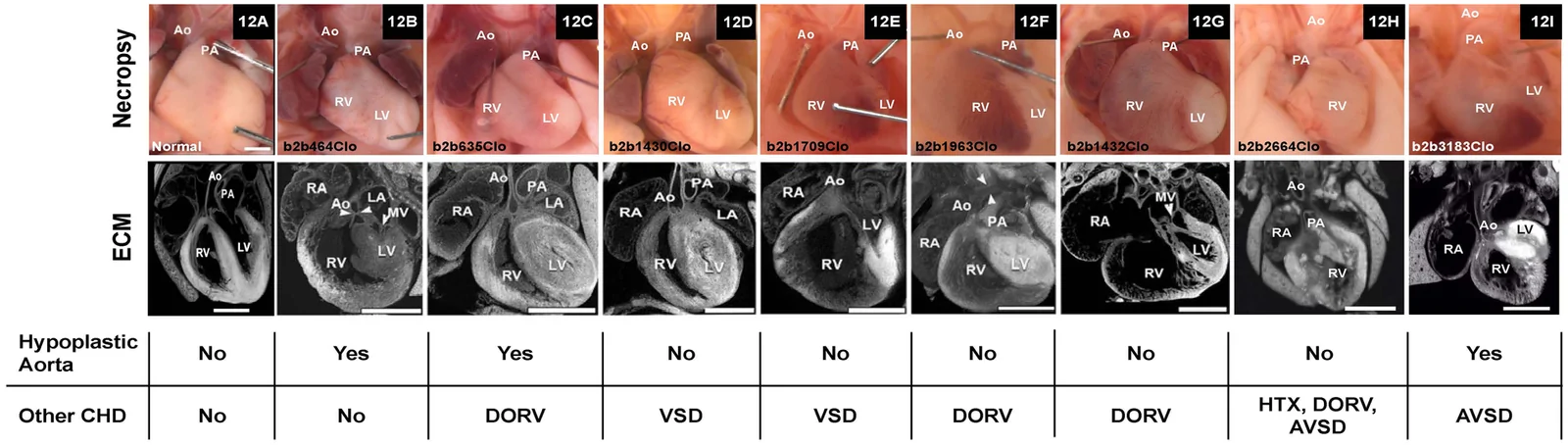
**Mouse models for HLHS**. Various degrees of hypoplastic left ventricle (LV) were observed in 8 HLHS mutant lines recovered from the mutagenesis screen. The top row shows necropsy photos, and the bottom row shows Episcopic confocal histopathology (ECM) of the same embryo. We observed a spectrum of left ventricular hypoplasia associated with ascending aortic hypoplasia in Line 464 (**B**), Line 635 (**C**), and Line 3183(I). A variety of congenital heart defects were observed in our HLV mouse models, such as double outlet right ventricle (DORV) in Line 635 **(C)**, Line 1963 **(F)**, Line 1432 **(G)**, and Line 2664 **(H)**. The embryos in Line 2664 (**H**) are associated with heterotaxy and atrioventricular septal defect (AVSD). Two mouse Lines are associated with small VSD: Line 1430 (**D**) and Line 1709 (**E**). Line 3183 (**I**) is associated with AVSD. HTX heterotaxy, MV mitral valve, LA left atrium, LV left ventricle, PA pulmonary artery, RA right atrium, RV right ventricle, TA truncal artery. [Modified with permission from Liu et al. (6)].

Analysis of phenotype/genotype in one HLHS mutant line, *Ohia* (Figure 12C), after extensive breeding identified a digenic etiology for HLHS, involving mutations in *Pcdha9*, a gene in the PCDHA gene cluster, and *Sap130*, which encodes a component of the histone deacetylase complex (HDAC) that regulates chromatin. Interestingly, some *Ohia* HLHS mutant mice also had a cardiac BAV phenotype. The observation that BAV can be observed in mutants harboring only the *Pcdha9* mutation, and small LV in mutants harboring only the *Sap130* mutation, suggests that the HLHS phenotype has a combinatorial digenic etiology. We also observed a very common *PCDHA* delCNV in the genetic etiology of BAV and HLHS described above (6, 135) from human studies.

### 4.5 HLHS-iPSC

In 2006, Dr Takahashi and Dr. Yamanaka discovered that differentiated somatic cells can be reprogrammed into an embryonic-like state, which is called induced pluripotent stem cells (iPSC) (153). IPS cells provide a strategy to study the complex genetics of HLHS. Our team and others used iPSC-derived cardiomyocytes to investigate the pathomechanisms of HLHS and observed mitochondrial and proliferation defects (154), impaired differentiation, lower beating rate, disorganized sarcomeres, and NOTCH1-dependent nitric oxide signaling abnormalities (154–159) in HLHS patients.

### 4.6 Abnormal NOTCH pathways result in the cardiac phenotype of left-sided lesions and/or a small LV

Insights into pathways such as NOTCH signaling, with identified loss-of-function variants like those in NOTCH1, open avenues for developing molecular therapies targeting these pathways in HLHS (126, 136–138, 156–162).

Studies with endothelial *Notch1/Rbpj* knockout demonstrated that Notch signaling in the endocardium is crucial for early ventricular chamber development by regulating cardiomyocyte proliferation and differentiation (163). In addition, we observed that one HLHS line (Line 1,430, Figures 12D) harbors Notch-related mutations (6). In HLHS mutant line 1430, we identified three Notch pathway mutations: homozygous mutations in *Nap1l1* and *Tspan14* and a heterozygous mutation in *Tspan15* (Figure 12D). *Nap1l1* was noted to regulate Notch1 activation and expression during the differentiation of iPSC-CM (164). *Tspan14 and 15* both regulate ligand-induced Notch signaling, with *Tspan14* providing positive regulation vs. *Tspan15* providing negative regulation of Notch signaling (165, 166). Consistent with our mouse model, Huang et al. used CRISPR-generated *Rbfox2*-deficient zebrafish and demonstrated an intrinsic myocardial defect (167). RBFOX2 is an RNA-binding protein that directly regulates the splicing of Numb, an intracellular regulator of the Notch signaling pathway (168).

### 4.7 Endocardial defects and endothelial-to-mesenchymal transition (Endo-MT) defects in HLHS

Miao et al. used single-cell RNA sequencing of human fetal heart tissue and iPSC-derived endothelial cells to identify abnormal functional pathways critical to valve formation and remodeling, including endothelial-to-mesenchymal transition (Endo-MT) defects, reduced extracellular matrix deposition, and reduced VEGF and NOTCH1 signaling. In addition, using an iPSC-induced endothelial cell-cardiomyocyte co-culture system, the team discovered that endocardial abnormalities in HLHS could cause cardiomyocyte proliferation defects, including abnormal cardiomyocyte maturation (169).

### 4.8 Microvascular senescence

Li et al. observed a senescent microvascular niche in the myocardium of HLHS patients. The microvascular niche includes endothelial cells, pericytes, and Yes-associated protein (YAP)-high fibroblasts, consistent with hypoxia and mechanical stress in HLHS. The team further demonstrated that these cellular and molecular changes can be reversed after ventricular assist device placement (170).

### 4.9 Abnormal cell proliferation, and cell death underlie LV hypoplasia

In the *Ohia* HLHS mouse model, the HLHS phenotype emerges very early during embryonic development. It can be detected as early as E11.5, before outflow tract septation is complete. Notable LV hypoplasia can be observed even in mutants with a near-normal-sized aorta, suggesting that cell-intrinsic defects may underlie LV hypoplasia in HLHS. Consistent with this, we observed a marked reduction in cardiomyocyte proliferation due to a metaphase block, primarily in the left ventricle, accompanied by increased apoptosis. Proliferation defects were also observed iPSC-CM generated from *Ohia* HLHS mouse lymphocytes and from HLHS patients. This suggests that proliferation defects and increased apoptosis reflect cell-intrinsic defects and are not dependent on hemodynamic perturbation (6, 154).

### 4.10 Myocardial defect, mitochondrial dysfunction, and uncompensated oxidative stress and heart failure

Despite clinical and medical advances allowing most HLHS patients to survive their critical congenital heart disease, there remains high morbidity and mortality from heart failure. Thus, up to 30% of HLHS patients die or survive with a heart transplant within the first year of life. As the causes of early heart failure are still poorly understood and donor organ supply is limited, heart transplant prioritization has been challenging. Analysis of *Ohia* HLHS mouse heart tissue, and mouse and human HLHS-iPSC-CM has revealed poor myocardial differentiation, accompanied by mitochondrial abnormalities, suggesting impaired contractile function exacerbated by mitochondrial bioenergetic deficits (154). This may contribute to the acute early heart failure risk in HLHS. These differentiation and mitochondrial respiration defects were accompanied by severe oxidative stress and endoplasmic reticulum (ER) stress, which were associated with increased apoptosis. Analysis of the iPSC-CM also showed reduced nitric oxide (NO) production, reminiscent of the previous finding of low nasal NO (nNO) in patients with HLHS, especially those exhibiting early heart failure (154).

In addition, we observed a defect in the mitochondrial transition pore in HLHS patients with early heart failure, which may directly influence mitochondrial permeability and cardiac function, highlighting a potential target for therapeutic intervention (154). Gene expression in the antioxidant pathway was greatly elevated in patients with favorable outcomes. In contrast, no changes or a reduction was observed in patients with early heart failure, suggesting early heart failure in HLHS may arise from uncompensated oxidative stress. Furthermore, we demonstrated that sildenafil and TUCA (a molecular chaperone that can suppress the unfolded protein response from oxidative/ER stress) can rescue the uncompensated oxidative/ER stress and increased apoptosis defects in early heart failure iPSC-CM (154). These compounds can restore mitochondrial permeability transition pore closure, respiration, and cell proliferation, and prevent apoptosis; however, their efficacy and safety require further validation in clinical settings before routine application (154).

### 4.11 Assessing mitochondrial function in peripheral blood provides a simple, noninvasive approach that could reassure clinicians regarding heart failure risk stratification in HLHS patients

We further demonstrated that mitochondrial dysfunction associated with the risk of heart failure in patients with HLHS can be readily assessed using oxygen consumption measurements in peripheral blood mononuclear cells (PBMCs) with the Seahorse Analyzer. This test provides a potentially simple, noninvasive method for heart failure risk stratification (171). Post-Fontan HLHS/single ventricle (SV) patients with heart failure showed higher respiratory maximum and reserve (Figures 13A–D). In contrast, these parameters were reduced in patients with HLHS/SV without heart failure (Figures 13A–D). We observed no difference in basal glycolysis between post-Fontan HLHS/SV patients, with or without heart failure, and biventricular (BV) CHD patients or normal control subjects. We observed the same results: elevated basal respiration (Figures 13E,F) in the *Ohia* mutants with HLHS and reduced LV basal respiration (Figure 13G) in the *Ohia* mutants without CHD (171). Together, these findings suggest intrinsic metabolic defects play a role in the risk of heart failure in HLHS (154, 171). While *in vitro* studies with iPS-CM showed decreased mitochondrial respiration associated with early heart failure, *in vivo* studies showed the opposite (154, 171). These paradoxical findings are likely reflect differences in hemodynamic forces between *in vivo* and *in vitro* conditions. Thus, *in vivo*, elevation in mitochondrial respiration may be a hemodynamically induced response compensating for the bioenergetic deficit associated with the mitochondrial respiration defect. In contrast, *in vitro*, without hemodynamic forces, intrinsic mitochondrial defects may be unmasked. These findings suggest that a rapid assessment of mitochondrial respiration using patient peripheral blood may provide a convenient method to determine the risk of heart failure in patients with HLHS. The elevated respiration associated with patients with early heart failure likely occurs in the context of a poor antioxidant response, thus exacerbating redox stress that can cause myocardial bioenergetic deficits and apoptosis that can cause early heart failure. These findings suggest some potential avenues for therapy to prevent HLHS-associated heart failure, such as pharmacological intervention to reduce redox stress or treatments that can improve the poor antioxidant response (154).

**Figure 13:**
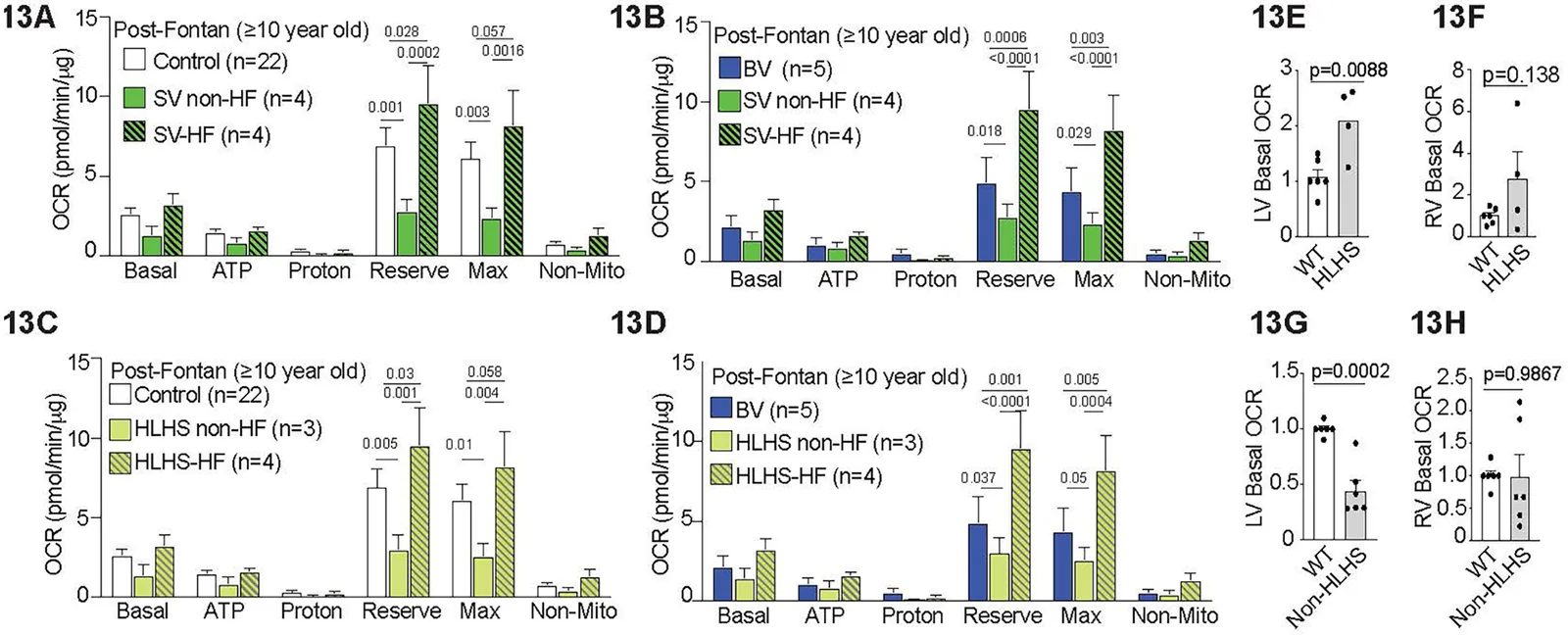
Mitochondrial respiration in SV-CHD patients and *Ohia* HLHS mutant mouse heart. **(A,B)** Mitochondrial respiration in the PBMCs of SV-CHD patients ≥10 years old with and without heart failure (HF) vs. age-matched controls **(A)** and BV-CHD patients **(B)**. **(C,D)** Mitochondrial respiration in the PBMCs of HLHS-CHD patients ≥10 years old with and without HF vs. age-matched controls **(C)** and BV-CHD patients **(D)**. **(E–H)** Basal oxygen consumption rate (OCR) in LV and RV heart tissue from E14.5-16.5 *Ohia* line with *Sap130/Pcdha9* mutations known to cause HLHS. This analysis was obtained using the Seahorse Analyzer. **(E,F)** Data were obtained from a litter comprising wild-type (WT) (*n* = 7) and HLHS (*n* = 4). **(G,H)** Quantitative analysis of 5 *Sap130/Pcdha9* mutants with normal cardiac anatomy **(E,F)** and six WT controls. **(A–D)** Mean ± SEM with one-way ANOVA test. Subject numbers are indicated in the graph legend. **(E–H)** Bar graphs show mean ± SEM with Student's *t*-test. Each dot represents one mouse embryo heart tissue [modified with permission from Xu et al. (171)].

**Figure 14:**
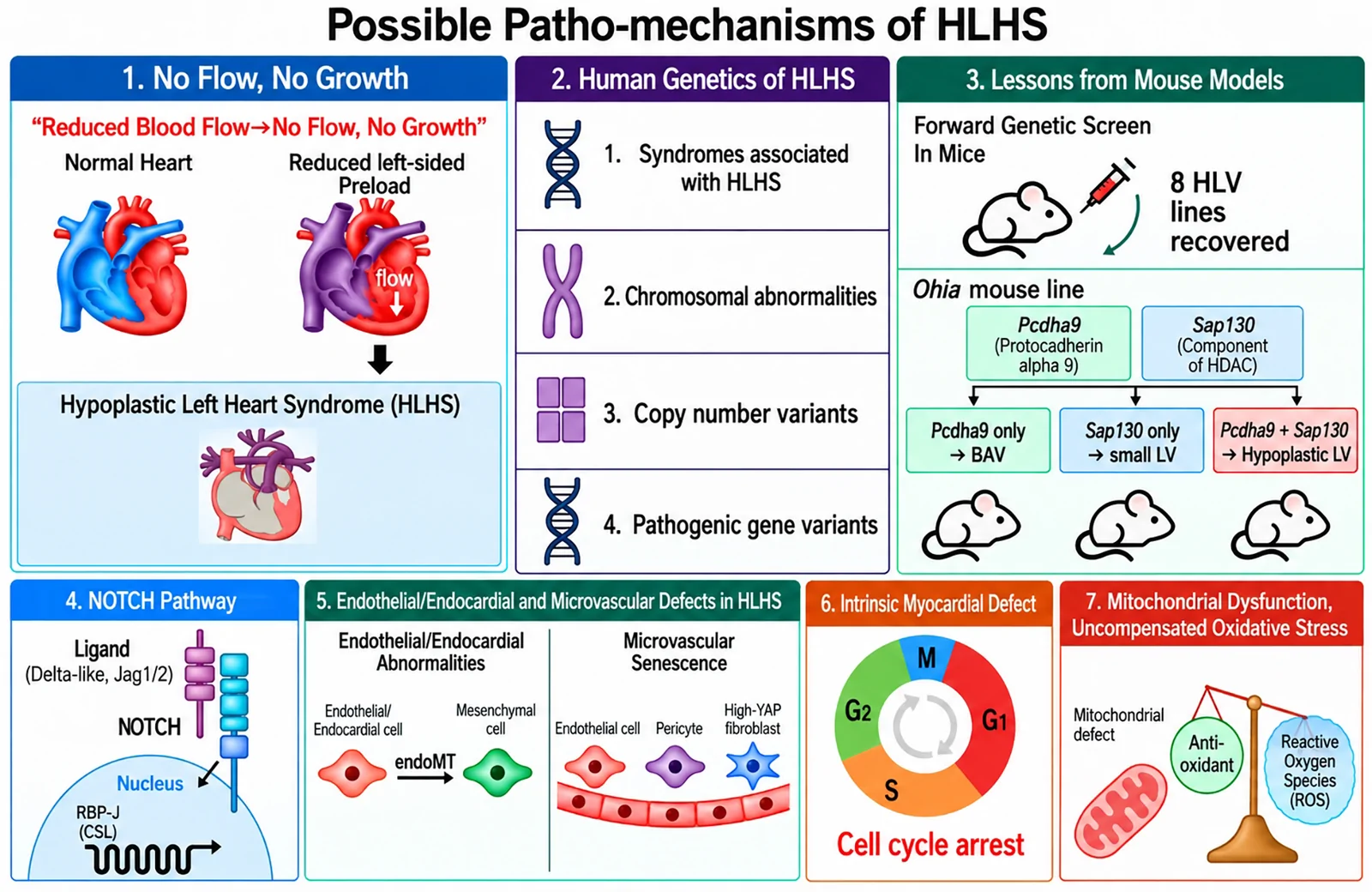
Illustration of the possible pathomechanisms of HLHS.

We summarize that the evidence supports HLHS as a **multifactorial disorder** involving abnormal hemodynamics, complex genetics, disrupted developmental signaling, intrinsic cardiomyocyte defects, and mitochondrial/metabolic dysfunction (Figure 14).

### 4.12 Stem cell therapies for HLHS

Because current standard care for HLHS has suboptimal outcomes, stem cell therapy, including IPSC therapy, offers an alternative strategy to deliver stem cells to the site of injury or lesion for repair and regeneration. Emerging clinical trials have demonstrated the promising potential of stem cell therapy (Table 1) (172). However, whether stem cell therapy can meaningfully improve long-term outcomes in HLHS warrants further investigation.

**Table 1:** Summary of stem cell clinical trials in HLHS.

Clinical Trial	Aims	Number of patients	Type of stem cells used	Mode of administration	Results
Ishigami et al. (2015) (173)	Phase 1 Whether intracoronary delivery of Cardiosphere-derived cells (CDCs) is feasible and safe (NCT01273857)	14 (7 received CDC injection, 7 controls)	Autologous Cardiosphere-derived cells	Intracoronary CDC infusion through heart catheterization 4–5 weeks after staged shunt procedures	Safe and feasible
Tarui et al., (2015) (174)	Assess safety and clinical outcomes of intracoronary infusion of CDCs (NCT01273857)	14	Cardiosphere-derived cells from the atrial cells harvested during stage I procedure	Intra-coronary CDC infusion 1 month after 2- or 3-stage surgical palliation	Improved RVEF in neonates receiving CDC with higher weight-for-age at 36 months and reduced BNP levels, and a lower incidence of unplanned catheter intervention.
Ishigami et al. (2017) (175)	Phase 2 Whether intracoronary delivery of CDCs improves cardiac function in single ventricle pathology	41	Autologous Cardiosphere-derived cells	Intracoronary	Improve heart failure status, somatic growth, and quality of life in patients and reduce parenting stress for the families.
Eitoku et al. (2018) (176)	Phase 1 and Phase 2 Transcoronary transfer of cells with stop-flow technique	Phase 1: 7 HLHS Phase 2: 41 patients with single ventricle pathology	Autologous Cardiosphere-derived cells	Intracoronary	The therapy was declared to be safe and feasible
Brizard et al. (2023) (177)	Phase 1: Safety and feasibility of adjunct autologous cord blood stem cell therapy (NCT03431480)	16	Autologous cord blood stem cell	Intracoronary during the Norwood procedure	Safe and feasible
Burkhart et al. (2015) (178)	Case report	1	Umbilical cord blood-derived mononuclear cells	Intramyocardial at the time of the stage II procedure	Improved RVEF during the 3-month follow-ups
Burkhart et al. (2019) (180)	Phase 1: Safety and feasibility of intraoperative umbilical cord blood-derived mononuclear cells	Phase 1: 10	Umbilical cord blood-derived mononuclear cells	Intramyocardial at the time of the stage II procedure	Safe and feasible, preservation of baseline RV function and normalization of growth rate
Kausal et al. (2023) (179)	Phase 1 (ELPIS-I): Whether Lomecel-B injection will be safe and feasible, or to report any new major adverse cardiac events (NCT03525418)	Phase 1: 10 HLHS received Lomecel-B Multipotent mesenchymal stem cells (MSCs) 4 HLHS received Allogenic MSC	Lomecel-B MSCs or allogeneic MSCs	Intramyocardial at the time of the stage II procedure	The therapy appears safe in HLHS patients and may favorably affect RV performance
Kausal et al.	Phase 2 (ELPIS-II) Test the hypothesis that Lomecel-B improves cardiac function compared to surgery alone (NCT04925024)	Targeted to enroll 38 HLHS patients	Lomecel-B MSCs	Intramyocardial at the time of the stage II procedure	Ongoing trial

BNP, brain natriuretic peptide; RV, right ventricle; RVEF, right ventricular ejection fraction. Modified from Choubey et al. (172).

## 5 Section IV: Neurodevelopment in HLHS

### 5.1 Association of adverse neurodevelopment with HLHS

Patients with complex CHD, especially those with HLHS, have been shown to have impaired neurodevelopment associated with developmental delay, learning disabilities, behavioral deficits, and cognitive impairment (181–189). The etiology of the cognitive impairment in HLHS had been generally assumed to arise from hypoxic injury before palliation and/or complications from medical/surgical management. Clinical studies in the Single Ventricle Reconstruction Trial analyzing various risk factors, including CPB time and hypothermia, showed that these factors explain only part of the variance in HLHS outcomes, suggesting that other intrinsic patient factors may contribute to poor neurodevelopmental outcomes in SV-CHD patients (181). As more individuals with HLHS survive adolescence and adulthood, it is more important than ever to help these patients not just survive, but also to thrive.

Children with HLHS are identified as being at the highest risk for developmental disabilities (187). The Single Ventricle Reconstruction compared outcomes after Norwood with modified BTT shunt versus Norwood with Sano shunt and followed these patients longitudinally, performing serial studies to assess neurodevelopmental outcomes. At age 14 months, both groups demonstrated lower Mental Development Index (MDI) and Psychomotor Development Index (PDI) scores on the Bayley Scales of Infant Development, 2nd edition (BSID-II) (188). The Single Ventricle Reconstruction Extension Study (SVR II) conducted annual developmental assessments using parents' questionnaires from ages 3 through 6 years. This study showed that the positive predictive values of the 14-month MDI and PDI for the 6-year Behavior Assessment System for Children, Second Edition (BASC-2) were low (44% and 36%, respectively). Nearly 29% of children with single-ventricle physiology scored in the at-risk or impaired range for adaptive skills, which is significantly higher than in the general population (188). The Single Ventricle Reconstruction-III trial (SVR III) invited the same cohort to participate in a comprehensive neurodevelopmental evaluation between ages 10 and 14. This study found these patients scored significantly lower than the normative population for intellectual and academic functioning, with more than one-third of individuals having significant deficits in learning, memory, attention/executive, social, emotional, behavioral, and adaptive functioning (189). Risk factors for worse neurodevelopmental outcomes included family socioeconomic status, longer total cardiopulmonary bypass time across surgeries, gastrostomy tube placement, and total complications through SVR III (189). Patients who underwent the Norwood procedure with a modified BTT versus a Sano shunt did not differ significantly in most neurodevelopmental scores (189). Other factors that contribute to long-term neurodevelopmental outcomes include concomitant genetic syndrome or abnormality, lower preoperative cerebral oxygenation, increased postoperative length of stay, preterm birth, microcephaly, and seizure history (187). Thus, early identification and intervention, along with ongoing multidisciplinary support, are critical in optimizing neurodevelopmental outcomes and improving quality of life for these patients.

### 5.2 Cortical brain abnormalities and neurobehavioral deficits in HLHS and epigenetic regulation

A large-scale human genomic study (PCGC) and studies of mutant mice with CHD have indicated a shared genetic etiology for cardiac defects and brain abnormalities (184, 190–194). These findings suggest that the specific mutations resulting in HLHS can also cause brain defects that can contribute to the poor neurodevelopmental outcome associated with HLHS. Studies in the HLHS mouse model showed similar mitotic defects in the developing brain (191) (Figures 15A–D,A’–D’) and heart (6) at the cell level. Microcephaly is observed in HLHS mice and has also been reported in HLHS patients (192, 193). In addition, forebrain structures, including the cerebral cortex, olfactory bulbs, hippocampus, corpus callosum, and cerebellum (Figures 15F, G, I, J), were affected in our mouse model as well as HLHS patients (191, 194). However, the midbrain and hindbrain structures were largely spared in patients with HLHS and in mice. These findings suggest regional brain dysplasia involving the forebrain may drive the neurodevelopmental delay and neurocognitive impairment associated with HLHS. We confirmed these findings using a mouse model in which *EMX-Cre* deletion of *Sap130* (a component of the digenic cause of HLHS in *Ohia* mutant mice) was performed in the forebrain. We observed microcephaly in the mutant mice, as well as abnormal behavior tests, including learning/memory deficits and autism like social interaction deficits (191).

**Figure 15:**
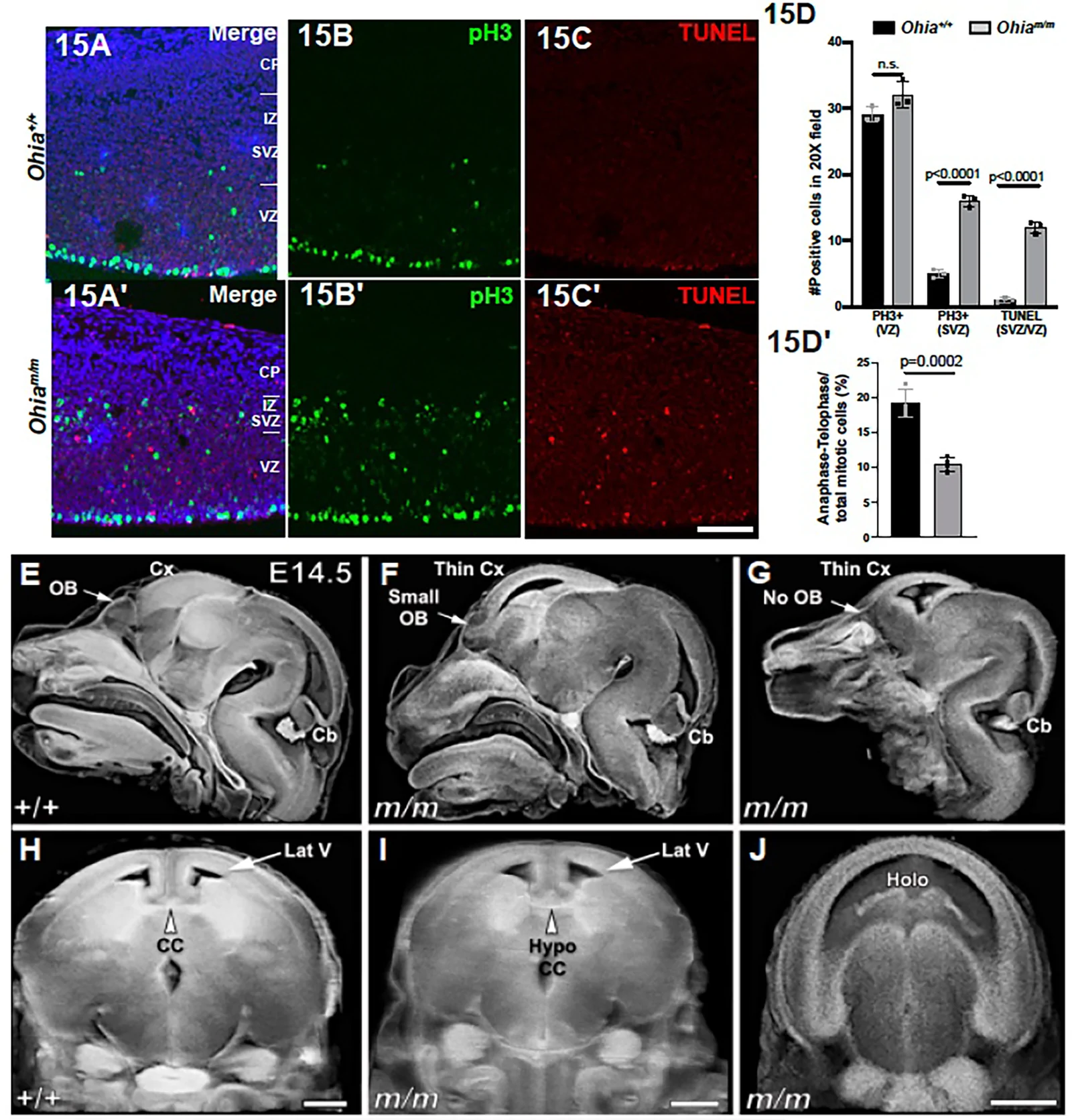
Brain anomalies in the HLHS mouse model. Compared to control animals **(A–C)**, the *Ohia* HLHS mutant **(A’–C’)** cortex shows more PH3 (indicating cells in M phase) and TUNEL staining (indicating apoptosis) localized to the ventricular (VZ) and subventricular (SVZ) zones at E14.5 [qualified in **(D, D’)**]. Episcopic confocal reconstruction in the sagittal plane of control **(E)**, an *Ohia* mutant with mild head defect **(F)**, and an *Ohia* mutant with severe head defect **(G)** shows that *Ohia* mutants (m/m) display hypoplastic forebrain, hypoplasia and thinning of the cerebral cortex (Cx), absent or hypoplastic olfactory bulb (OB), abnormal folding/shape of cerebellum (Cb), hypoplasia of choroid plexus, and hippocampal dysplasia. Reconstruction in the coronal plane of control **(H)**, an *Ohia* mutant with mild head defect **(I)**, and an *Ohia* mutant with severe head defect **(J)** shows that *Ohia* mutants also display holoprosencephaly (Holo) and corpus callosum (CC) defects. Lat V: lateral ventricle. [modified with permission from Gabriel et al. (191)].

Transcriptome profiling of the *Ohia* HLHS mouse brain and heart by RNA-seq showed that two of the top enriched pathways were “metabolic pathway” and “axon guidance”. A significant perturbation of many genes associated with intellectual disability and learning and memory deficits in the brain RNAseq was observed (191). These findings suggest regional brain dysplasia may drive the neurodevelopmental delay associated with HLHS. A role for mitochondrial dysfunction is also suggested by the recovery of metabolic pathways (154).

As the CHD and microcephaly phenotypes showed incomplete penetrance in the *Ohia* HLHS mouse model, we investigated possible epigenetic regulation by analyzing genome-wide DNA methylation. Using a whole-mouse-genome DNA methylation microarray, we observed marked changes in DNA methylation associated with genes implicated in microcephaly, intellectual disability, and cognitive impairment, as well as a range of neurodevelopmental and neurobehavioral abnormalities. These findings indicate a critical role for epigenetic regulation in determining the penetrance of adverse neurodevelopment in HLHS (191). Epigenetic involvement also suggests new therapeutic avenues, as interventions that affect DNA methylation can modify the epigenome. Such therapy provided postnatally should be explored for the potential to ameliorate neurobehavioral and neurocognitive impairments associated with HLHS.

## 6 Conclusion

HLHS is a CHD with significant morbidity and mortality. While surgical and medical advances have increased life expectancy, resulting in an ever-growing population of adult HLHS patients, there remain significant challenges with heart failure, liver disease associated with post-Fontan surgical complications, and neurocognitive/neurobehavioral impairments. Integration of research from human and mouse models suggests that HLHS results from complex genetic etiologies that cause cell-intrinsic metabolic and myocardial differentiation defects, accompanied by hemodynamic derangements. The finding of convergence in the abnormal cellular and molecular defects in the heart and brain suggests new avenues of therapy may have benefit for both heart failure and the adverse neurocognitive/neurobehavioral outcomes. Together, these *in vivo* and *in vitro* findings in mice and humans uncovered new therapeutic targets for HLHS that may pave the way for therapies to improve cardiac and neurodevelopmental outcomes.
